# Enhanced photoelectrical response of thermodynamically epitaxial organic crystals at the two-dimensional limit

**DOI:** 10.1038/s41467-019-08573-8

**Published:** 2019-02-14

**Authors:** Min Cao, Cong Zhang, Zhi Cai, Chengcheng Xiao, Xiaosong Chen, Kongyang Yi, Yingguo Yang, Yunhao Lu, Dacheng Wei

**Affiliations:** 1 State Key Laboratory of Molecular Engineering of Polymers, Fudan University, Shanghai, 200433 China; 2 Department of Macromolecular Science, Fudan University, Shanghai, 200433 China; 3 International Center for New-Structured Materials and School of Materials Science and Engineering, Zhejiang University, Hangzhou, 310027 China; 4 Shanghai Institute of Applied Physics, Chinese Academy of Sciences, 2019 Jialuo Road, Shanghai, 201800 China

## Abstract

Owing to strong light-matter interaction, two-dimensional (2D) organic crystal is regarded as promising materials for ultrasensitive photodetectors, however it still received limited success due to degraded photoelectrical response and problems in controllable growth. Here, we find the growth of 2D organic crystal obeys Gibbs-Curie-Wulff law, and develop a seed-epitaxial drop-casting method to grow millimeter-sized 1,4-bis(4-methylstyryl)benzene 2D crystals on SiO_2_/Si in a thermodynamically controlled process. On SiO_2_/Si, a distinct 2D limit effect is observed, which remarkably enhances internal photoresponsivity compared with bulk crystals. Experiment and calculation show the molecules stack more compactly at the 2D limit, thus better molecular orbital overlap and corresponding changes in the band structure lead to efficient separation and transfer of photo-generated carriers as well as enhanced photo-gating modulation. This work provides a general insight into the growth and the dimension effect of the 2D organic crystal, which is valuable for the application in high-performance photoelectrical devices.

## Abstract (web summary)

To realize efficient optoelectronic devices based on two-dimensional (2D) organic crystals, optimizing the photoelectrical response and growth of these materials at the 2D limit is vital. Here, the authors report enhanced internal photoresponse in large-area 2D crystals using a novel growth method.

## Introduction

Two-dimensional (2D) materials are normally regarded as a promising material in future ultrasensitive photodetectors, owing to their strong light–matter interaction at the 2D limit^1,2^. Besides well-known 2D atomic crystals (2DACs), 2D organic crystals (2DOCs) have emerged as a new class of prospective 2D materials for photoelectrical applications, which bring advantages, such as low-cost solution processability, superior mechanical flexibility, tailored band structures, and device performances by ingenious molecular design^3,4^. Pioneer research on 2DOCs indicates that the molecular packing and the resulting charge transport at the 2D limit is considerably different from that in the bulk^5–9^. Electrical devices, such as field-effect transistors^6–12^ and chemical sensors^13^ based on 2DOCs, exhibit a performance comparable or higher than that of their bulk counterparts, as a result of efficient charge carrier injection and high surface-to-volume ratio owing to their 2D feature. Although tremendous developments have been achieved in electrical devices, their applications in photoelectrical devices have met with limited success, which is partially attributed to the weak light absorbance due to their molecular-scale thickness, as well as the absence of a clear understanding of the relationship between the photoelectrical behavior and the crystalline structure of an organic crystal at the 2D limit. 2DOCs are bonded via van-der-Waals interaction, which is much weaker than the covalent bonds in 2DACs^6^. As a result, the crystalline structure and the properties of an organic crystal can be easily tuned^14,15^, especially at the 2D limit. When the dimension decreases from bulk to one to several molecular layers, the molecule–substrate interaction becomes non-ignorable, which normally leads to face-on or a largely tilted molecular packing configuration^6,7,10^, hampering the separation and transfer of photo-generated carriers. Therefore, organic crystals have a different 2D limit effect from 2DACs, which normally degrades the photoelectrical performance to a level much lower than that of bulk organic crystals or thin films, resulting in limited success of 2DOCs in various photoelectrical applications.

As another obstacle for the photoelectrical application, large-area high-quality 2DOCs need be produced in a controllable manner. So far, besides physical vapor deposition^6,10^, solution-based techniques, including solution epitaxy at the solution/air interface^7^, drop-casting^16^, and floating-coffee-ring-driven assembly method^8,17^ have been developed to grow 2DOCs with sizes up to millimeters. However, the crystallization via weak van-der-Waals force is sensitive to external conditions^14^, and it is still a challenge to produce large-sized 2DOCs with controllable thickness inside the solution, especially for the organic crystals with small surface energy difference for each crystal facet. Until now, the inherent principles of crystal nuclei evolution to large-sized 2DOCs inside the solution have not been clarified, leading to problems in controllable growth and the lack of fundamental understanding on how to promote the 2D growth of organic crystals by certain crystallization conditions.

Here, we find that the epitaxial growth of 2DOCs inside the solution obeys Gibbs–Curie–Wulff law, which requires thermodynamically controlled conditions, such as relatively high temperature, oversaturated solution with high boiling solvent, and seed crystals, in order to accelerate the lateral 2D growth and reach a surface energy minimum at equilibrium. Based on this finding, we develop a simple seed-epitaxial drop-casting method to grow millimeter-sized 1,4-bis(4-methylstyryl)benzene (p-MSB) 2D single crystals on thermally oxidized SiO_2_ on Si (SiO_2_/Si) substrate with relatively controllable thickness. More importantly, a distinct 2D limit effect is observed on SiO_2_/Si, which remarkably enhances the photoelectrical response. Measurement and calculation show that the molecules are inclined to stack more compactly on SiO_2_/Si with decreasing crystal thickness, owing to their weak interaction with SiO_2_/Si and the strong intra-layer π-stacking. As a result, the molecular orbitals overlap horizontally to form a continuous network, which contributes to delocalized carriers, changes in the band structure, and band-like excimer photoluminescence (PL) emission. This, as well as enhanced photo-gating modulation, gives rise to a remarkable increase of the internal photoresponsivity (*R*_in_) up to 2.13 × 10^5^ A W^−1^ for 254-nm light and 3.28 × 10^5^ A W^−1^ for 365-nm light at the 2D limit, about 2–3 orders higher than those of bulk crystals. The external photoresponsivity (*R*_ex_) reaches 2.74 × 10^4^ A W^−1^ under 254-nm illumination, comparable or higher than that of most phototransistors based on 2DACs (i.e., 880 A W^−1^ for MoS_2_, 19.1 A W^−1^ for GaS, 10^2^ A W^−1^ for SnS_2_, and 657 mA W^−1^ for black phosphorus)^18–21^. Despite their ultrathin thickness, the *R*_ex_ is still among the best results of ultraviolet phototransistors based on organic single crystals or organic thin films (10^3^–10^4^ A W^−1^)^15,22–26^. Besides the 2D p-MSB crystal, similar enhanced photoresponse is also observed in perylene or α-sexithiophene (α-6T) 2D crystals, showing the great potential of 2DOCs in high-performance photoelectrical devices.

## Results

### Growth and structure of 2D p-MSB crystals

**Fig. 1:**
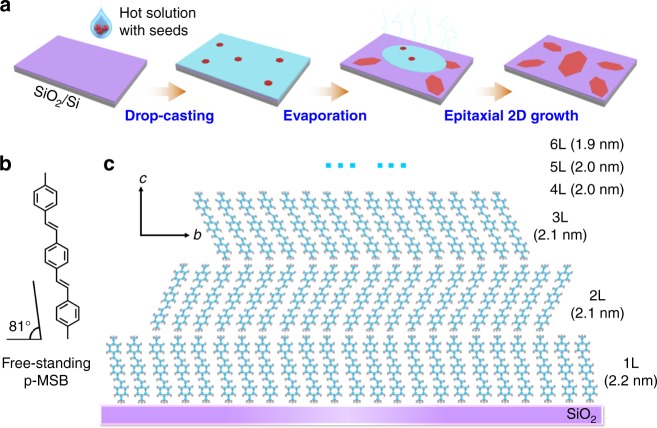
Growth of two-dimensional (2D) p-MSB crystals. **a** Schematic illustration of the drop-casting growth process. **b** A freestanding p-MSB molecule. **c** Schematic illustration of the molecular packing of 1 L, 2 L, and 3 L on SiO_2_/Si within the *b–c* plane

**Fig. 2:**
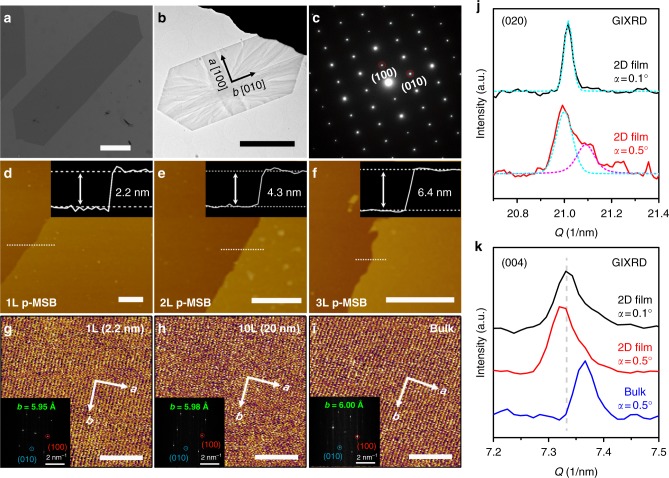
As-grown 2D p-MSB crystal. **a** Scanning electron microscope (SEM) image of a typical millimeter-sized 2D p-MSB crystal grown on SiO_2_/Si. **b** Transmission electron microscope (TEM) image and **c**, the selected-area electron diffraction (SAED) analysis of a 2D p-MSB crystal. **d**–**f** AFM images of monolayer (1 L), bilayer (2 L), and trilayer (3 L) p-MSB on SiO_2_/Si, respectively. The insets are the corresponding height profiles across the dashed white lines in **d**–**f**. **g**–**i** High-resolution atomic force microscope (AFM) images of the monolayer (2.2 nm), thin-film (20 nm), and bulk p-MSB crystals on SiO_2_/Si. The insets are the corresponding fast Fourier transforms (FFTs) of the high-resolution AFM images. **j**, **k** Grazing incident X-ray diffraction (GIXRD) patterns of the 2D p-MSB or bulk p-MSB film for (020) and (004) as a function of scattering vector (*Q*), measured at different incidence angles (*α* = 0.1° or 0.5°). The scale bars in **a**, **b**, **d**–**f**, **g**–**h** are 200 μm, 5 μm, 1 μm, and 10 nm, respectively

Two-dimensional p-MSB crystals were prepared by a seed-epitaxial drop-casting method (Fig. 1). Scanning electron microscopy (SEM, Fig. 2a) and transmission electron microscopy (TEM) images (Fig. 2b, Supplementary Fig. 1) show that the p-MSB crystals have well-defined elongated hexagonal shapes with 1.1-mm length. Some crystals have a length even up to 2.5 mm (Supplementary Fig. 2). Selected-area electron diffraction (SAED) patterns (Fig. 2c, Supplementary Fig. 1) contain a single set of diffraction spots, which are indexed to (010) and (100) crystal planes. This, as well as uniform light extinction in the cross-polarized optical microscope image (Supplementary Fig. 3), indicates the single-crystalline nature and the fact that the p-MSB crystal grows along [010] and [100] directions in its a–b crystal plane parallel to the substrate^27^. Some stripe-like contrasts are observed in the TEM image, which are attributed to the strain originating from the bending of the crystal^28^, indicating the high flexibility of the 2D p-MSB crystal.

We studied the crystalline structure of the 2D p-MSB crystals on SiO_2_/Si. Atomic force microscopy (AFM, Fig. 2d–f) images show that the crystals are uniform and atomically flat. The thickness of the initial four layers is about 2.2 nm, 2.1 nm, 2.1 nm, and 2.0 nm (Supplementary Table 1, Supplementary Fig. 4), respectively, while the average thickness of each molecular layer in the bulk is 1.9 nm^27^, indicating that the tilt angles and the molecular packing in the initial few layers are different from those in the bulk. The height profiles from single 2D crystals (Supplementary Fig. 5) clearly show that the bottom layers are thicker than the upper layers; thus, the p-MSB molecules are standing more vertically on SiO_2_/Si, when the crystal thickness decreases to 2D dimension (Fig. 1c). In the case of monolayer p-MSB, the molecule is almost uprightly standing on SiO_2_/Si with an immanent tilt angle of 9° (Fig. 1b), since the p-MSB molecule is asymmetric.

The detailed crystalline structure was studied by high-resolution AFM, TEM, and synchrotron radiation grazing incident X-ray diffraction (GIXRD). High-resolution AFM images and the corresponding fast Fourier transform (FFT) patterns (Fig. 2g–i, Supplementary Figs. 6–8) of the bulk (~120 nm), thin-film (~20 nm), and monolayer (2.2 nm) crystals on SiO_2_/Si reveal a shrink along the b axes (from 6.00 ± 0.02 Å to 5.95 ± 0.02 Å). SAED studies give a similar result (Supplementary Fig. 9), that the b/a ratio decreases from 0.782 for the bulk to 0.774 for the 2D crystal, indicating the more compact π–π stacking structure with decreasing crystal thickness on SiO_2_/Si. GIXRD data (Fig. 2j, k) confirm the above-discussed revolution of the crystalline structure. In the measurement, the X-ray penetration depth is determined by the incidence angle (*α*), thus allowing precise characterization of the crystalline structure at different depths^29^. With increasing α from 0.1° to 0.5°, a new (020) sub-peak of the 2D p-MSB film appears at 21.09 nm^−1^, indicating that the bottom layers are stacked more compactly, compared with the upper layers, while the (004) peak shifts negatively, indicating that the bottom layers are thicker than the upper layers. At the same α (0.5°), the (004) peak of the bulk sample positively shifts to about 0.04 nm^−1^, showing the relatively smaller layer thickness compared with the 2DOCs.

### Thermodynamical epitaxial growth mechanism

**Fig. 3:**
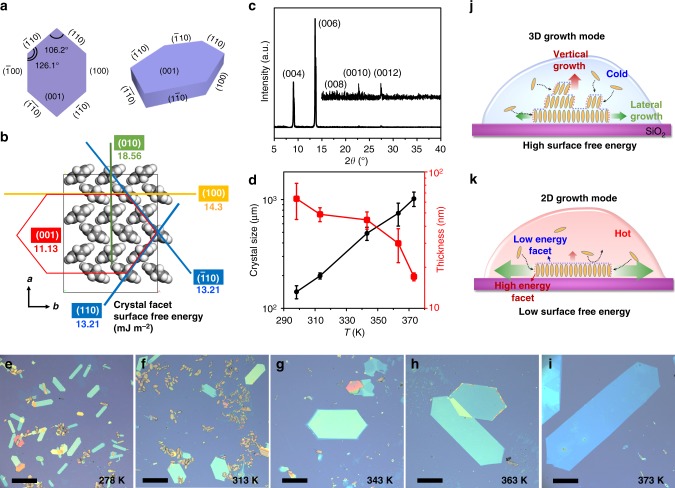
Thermodynamical epitaxial growth of 2D p-MSB crystals. **a** Schematic diagrams of a p-MSB single crystal with indexed facets viewing along the *c* axes and at about 45° angle with respect to the *c* direction. **b** Molecular arrangement and **c** X-ray diffraction (XRD) pattern of the p-MSB crystal. The calculated surface-free energy of each crystal facet is shown in **b**. **d** Plots of average crystal size and average thickness with respect to the growth temperature (all crystal sizes are quantified using the diagonal length of the crystals). **e**–**i** Optical images of p-MSB crystals grown at different temperatures from 298 K to 373 K. **j**, **k** Growth mechanism of two different growth modes. The ratio of the lateral growth rate to the vertical growth rate varies with temperatures. High-energy facet and low-energy facet are indicated by red and blue dashed lines, respectively. The thermodynamically controlled 2D growth mode can reach a surface energy minimum. The scale bars in **e**–**i** are 200 μm. The error bars in **d** represent the standard error of the mean (s.e.m.) of the average size or the average thickness of the crystal measured from the optical or AFM images

To clarify the growth mechanism, we captured the growth process by an optical microscope (Supplementary Note 1, Supplementary Fig. 10, Supplementary Movie 1). The result shows that the growth mainly takes place on the substrate inside the toluene solution. The crystal size gradually increases along with the solvent evaporation without obvious contrast change, indicating a 2D growth mode inside the solution. Although actual solution-based growth is complicated^14^, Gibbs–Curie–Wulff law has been proved to be a reliable guidance on organic crystal engineering^30^. Gibbs argues that a crystal at a thermodynamical equilibrium should, in principle, adopt a shape of minimum surface-free energy; thus, the shape is highly dependent on the surface-free energy of the corresponding crystal facets (Fig. 3a, b). In X-ray diffraction (XRD) pattern (Fig. 3c) of the p-MSB crystal, only (00k) reflections are observed, indicating a layer-by-layer growth mode along the [001] axes perpendicular to the substrate. According to the law of constancy of interfacial angles, the faceted angles of 106.2° and 126.1° in (001) are between the lateral (100), (110), (−110), (−100), (−1−10), and (1−10) facets, as depicted in Fig. 3a. The density functional theory (DFT) calculation (Fig. 3b) shows that the (001) facet has the lowest surface-free energy compared with other lateral planes. Therefore, according to Gibbs–Curie–Wulff law, p-MSB molecules prefer a 2D growth mode and the largest facet should be (001) in a thermodynamical equilibrium. However, the crystallization of organic molecules via weak van-der-Waals force is sensitive to external conditions (Supplementary Note 2, Supplementary Fig. 11). Compared with other molecules (like DBTDT) reported previously^30^, the p-MSB crystal has a much smaller difference of the surface-free energy for each crystal facet (Fig. 3b), leading to an undesired 3D growth mode as well as uncontrollable thickness when the growth takes place inside the solution. It is possible to produce crystalline p-MSB films via existing technologies, such as solution epitaxy^7^, self-assembly driven by the coffee-ring effect^8,17^, in which the growth takes place on the solution surface or at the edge interface of the droplet (Supplementary Note 3, Supplementary Fig. 12 and 13). To achieve the 2D growth of p-MSB inside the solution without the aid of these interfaces, controlled experiments show that a thermodynamically controlled growth process is required. In such a process, a steady and appropriate crystallization rate is pivotal, which requires relatively high temperature, oversaturated solution with high boiling solvent, and seed crystals.

We prepared p-MSB crystals inside the solution at different temperatures (Supplementary Note 4, Supplementary Fig. 14). If the growth takes place in an ice bath (≤ 273 K), only small thick crystals are obtained. As the temperature gradually increases from room temperature (298 K) to 373 K (Fig. 3d–i), the average crystal size (Supplementary Fig. 15) increases from ~110 μm to ~1 mm, while the average thickness (Supplementary Fig. 16) decreases from ~65 nm to ~17 nm, indicating that the growth varies from 3D mode toward 2D mode at a higher temperature. As a result of a small difference of the surface-free energy for each crystal facet (Fig. 3b), the growth rate differentiation of p-MSB exhibits a stronger dependence on the temperature. At low temperature, epitaxial growth occurs along with random nucleation in the bulk solution. Both lateral and vertical growth rates are very slow, resulting in inhomogeneous, small, and thick crystals (Fig. 3j, Supplementary Fig. 14d). At a higher temperature, both the dissolution and the crystallization rate increase, which remarkably suppresses the nucleation and significantly increases the lateral growth rate when compared with the vertical growth rate. A thermodynamical equilibrium shape with a surface energy minimum is expected, showing a 2D growth mode (Fig. 3k)^31,32^. Thus, the residues and thick crystals decrease with increasing temperature. At a temperature higher than 353 K (Fig. 3h, i, Supplementary Fig. 14a), the main products grown inside the solution are 2DOCs with high-yield formation. Besides p-MSB, we also produce perylene or α-6T crystals by the seed-epitaxial drop-casting method. Larger and thinner crystals (Supplementary Fig. 17) are obtained at 353 K compared with those grown at room temperature.

Besides the temperature, other factors also influence the growth of the 2DOCs^14^, such as the molecular concentration, the coffee-ring effect (Supplementary Note 5)^7,33^, the boiling point, viscosity, and surface tension of the solvent (Supplementary Note 3)^34^. For instance, p-MSB crystals nucleate and grow not only from the seed crystal in the solution, but also at the edge of the droplet or the substrate owing to the coffee-ring effect (Supplementary Fig. 18, 19, Supplementary Movie 2). In the case of the growth inside the solution, the oversaturated solution with high boiling solvent and seed crystals is of great importance. Toluene has a high boiling point; thus, evaporation of the solvent can be well controlled, offering a steady and appropriate crystallization rate for molecules to assemble into thermodynamically stable morphologies^35^. In the case of low-boiling-point solvents, such as dichloromethane and acetonitrile, the solvent evaporates rapidly, resulting in large amounts of small crystals or nuclei (Supplementary Fig. 20). The seed crystal (Supplementary Note 6, Supplementary Fig. 21) also offers a stable epitaxial crystallization process. Otherwise, with an unsaturated or saturated solution without seed crystals, no 2D p-MSB crystals are nucleated and grown inside the solution, while concentric rings of flakes are obtained and maintained as concentration gradually increased up to saturation, owing to the coffee-ring effect and the repeated stick-slip motion of contact line (Supplementary Fig. 22, Supplementary Movie 3)^33^.

### Photoelectrical properties of 2D organic crystals

**Fig. 4:**
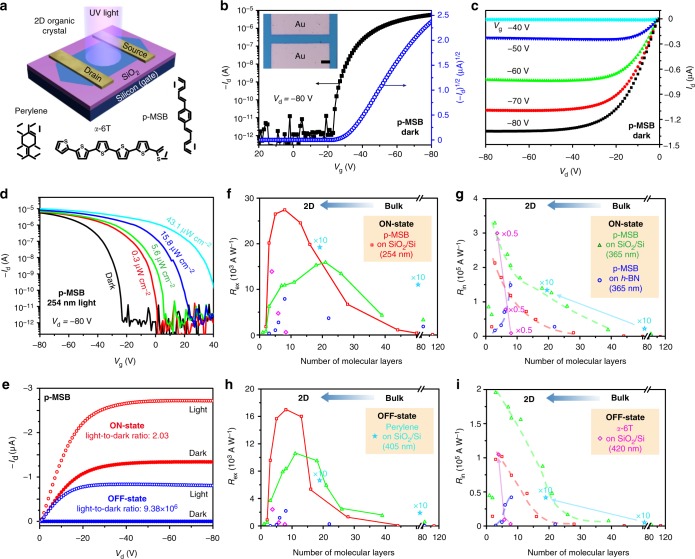
Electrical and photoelectrical characteristics of the crystals. **a** Bird’s-eye view of a schematic configuration of the photodetector based on a 2D organic crystal, and the molecular structures of p-MSB, perylene, and α-6T. **b** Transfer (drain current (*I*_d_) vs. gate voltage (*V*_g_), when drain voltage (*V*_d_) is −80 V) and **c** output (*I*_d_ vs. *V*_d_) curves of a typical 2D p-MSB device. The inset of **b** is the optical microscopy image of a 2D p-MSB device. **d** Transfer curves of the device (*V*_d_ = −80 V) in the dark or under 254-nm illumination with different power intensities. **e** On-state (*V*_g_ = −80 V) and off-state (*V*_g_ = −20 V) *I*_d_ versus *V*_d_ curves of the p-MSB device in the dark or under 254-nm illumination (43.1 μW cm^−2^). **f**, **h** External photoresponsivity (*R*_ex_) and **g**, **i**, internal photoresponsivity (*R*_in_) as a function of the number of molecular layers (p-MSB, perylene, or α-6T) under illumination (12.5 μW cm^−2^ 254 nm, 14.1 μW cm^−2^ 365 nm, 112 μW cm^−2^ 405 nm, and 141 μW cm^−2^ 420 nm) when the devices operate in the ON-state (**f**, **g**) or OFF-state (**h**, **i**). The dashed lines in **g** and **i** indicate different 2D limit effects on the *R*_in_ when the crystal is located on different substrates (SiO_2_/Si or *h*-BN). The scale bar in **b** is 20 μm

Electrical devices were fabricated on 2DOCs by thermal deposition (Fig. 4a). The transfer characteristics of p-MSB (Fig. 4b) exhibit a typical p-type semiconductor behavior with on/off current ratio up to ~10^6^. The output curves are linear at low drain voltage (*V*_d_) without S-shaped characteristics, and such linear behavior (Fig. 4c) indicates good charge injection^36^. We measured more than 30 devices, and the hole mobilities are 0.004–0.226 cm^2^ V^−1^ s^−1^ depending on the thickness, comparable with or even higher than the best reported result (0.17 cm^2^ V^−1^ s^−1^)^37^ or our result (0.022 cm^2^ V^−1^ s^−1^, Supplementary Fig. 23) of the p-MSB single crystal grown by physical vapor transport (PVT), showing the high sample quality. Hole mobility of the p-MSB crystal along [010] is about 3.8 times higher than that along [001] (Supplementary Fig. 24). Such an anisotropic transport is attributed to the π–π stacking direction along [010], suggesting that the *b* axis is the main carrier transport direction.

Owing to the strong absorption of the ultraviolet light (Supplementary Fig. 25), the devices show significant response to the incident light only when the wavelength is shorter than 420 nm (Supplementary Fig. 26), indicating the potential application in visible–blind ultraviolet detection. Figure 4d shows the transfer curves (*V*_d_ = −80 V) under 254-nm light with different power intensities. With increasing incident power intensity, the photocurrent increases, while the threshold voltage (*V*_th_) shifts positively, indicating that the photoresponse arises from the synergy of photoconduction and photo-gating effects (Supplementary Note 7)^15,38–40^. Under an electrical-gating modulation^41^, the device in the OFF-state of the transistor (*V*_g_ = −20 V) has high light-to-dark current ratio up to 9.38 × 10^6^ (Fig. 4e), six orders of magnitude higher than that (2.03) obtained in the ON-state (*V*_g_ = −80 V).

Both the electrical and photoelectrical properties have a strong dependence on the layer number of the p-MSB crystals. Here, the *R*_ex_ is defined as the ratio of the photocurrent and the incident light power^42^, while the *R*_in_ represents the ratio of the photocurrent and the absorbed light power^43^. As a result of the fact that a thicker crystal absorbs more photons, the *R*_ex_ (in the ON-state of the transistor, *V*_g_ = −80 V) increases with increasing layer number from a monolayer to nine layers (12.5 μW cm^−2^ 254-nm light) or 21 layers (14.1 μW cm^−2^ 365-nm light), and then reaches saturation owing to the limited exciton diffusion length (Fig. 4f). The highest *R*_ex_ reaches 2.74 × 10^4^ A W^−1^ for 254-nm light (*V*_d _= −80 V) and 1.59 × 10^4^ A W^−1^ for 365-nm light (*V*_d _= −80 V), which is among the highest results of organic thin-film phototransistors (10^3^–10^4^ A W^−1^) despite the ultrathin thickness^15,22,25,26,44^. The photodetectivity (D*) which describes the smallest detectable signal (calculated by D* = (*R*_ex_ *S*^1/2^)/(2e *I*_d_)^1/2^, where *S* is effect area of the photodetector, e is an electron charge, and *I*_d_ is the dark current) is 7.8 × 10^13^ Jones (254-nm light) and 4.5 × 10^13^ Jones (365-nm light) for nine layers. The *R*_ex_ and D* values are larger than those of bulk crystals (89 A W^−1^, 2.7 × 10^11^ Jones for 254-nm light; 3.3 × 10^3^ A W^−1^, 1.0 × 10^13^ Jones for 365-nm light) produced by drop-casting method, which is probably attributed to the inherent photoelectrical properties of the organic 2D crystal as well as the improved contact between the crystal and the electrodes owing to the thin channel thickness^9,12^. To reflect the inherent photoelectrical properties and to exclude the influence of thickness on light absorption, *R*_in_ is calculated by *R*_in_ = *R*_ex_/*A*, where *A* = 1 − exp (−*αx*) is the absorbance of the p-MSB crystal with thickness *x* and absorption coefficient *α* (Supplementary Note 8, Supplementary Fig. 27)^45^. The *R*_in_ (in the ON-state of *V*_g_ = −80 V) increases with decreasing layer number (Fig. 4f), and the maximum *R*_in_ is 2.13 × 10^5^ A W^−1^ for 254-nm light (*V*_d _= −80 V) and 3.28 × 10^5^ A W^−1^ for 365-nm light (*V*_d _= −80 V) for 3 L (the small *R*_in_ for 1 L and 2 L is attributed to the interfacial defects and the influence of the amorphous surface of SiO_2_/Si, as a result of the ultrathin thickness), more than one order higher than that of bulk crystals (1.01 × 10^2^ A W^−1^ for 254- nm light; 7.85 × 10^3^ A W^−1^ for 365-nm light). These values are also higher than *R*_in_ (6.63 × 10^3^ A W^−1^ for 254-nm light), *R*_ex_ (6.63 × 10^3^ A W^−1^ for 254-nm light), and D* (1.91 × 10^13^ Jones) of the bulk single crystal (459-nm thick) produced by PVT (Supplementary Fig. 23), showing the high sample quality and the enhancement in photoelectrical response at the 2D limit. Moreover, we also measured *R*_ex_ and *R*_in_ of the p-MSB crystal in the OFF-state of the transistor (*V*_g_ = −20 V). Owing to the decreased photo-gating contribution to the total photocurrent^41^, the values (Fig. 4h, i) are smaller than those obtained in the ON-state of the transistor. Nevertheless, similar thickness-dependent behavior is observed. The *R*_in_ increases with decreasing layer number, and the maximum *R*_in_ is obtained when the layer number is 3–4 L (1.01 × 10^5^ A W^−1^ for 254-nm light; 1.95 × 10^5^ A W^−1^ for 365-nm light). The corresponding external (EQE) and internal quantum efficiency (IQE) can be calculated as QE = *R E*_ph_/*q*, where *q* is the electron charge, *E*_ph_ is the photon energy, and *R* is *R*_ex_ or *R*_in_, respectively. The EQE and IQE exhibit the same trend as the photoresponsivity (Supplementary Fig. 28).

Besides p-MSB, we also produced phototransistor devices using perylene (Supplementary Fig. 29) or α-6T (Supplementary Fig. 30) crystals. The *R*_ex_, *R*_in_, and D* of perylene increase from 1.16 × 10^3^ A W^−1^, 2.43 × 10^3^ A W^−1^, and 4.54 × 10^12^ Jones to 1.95 × 10^3^ A W^−1^, 1.33 × 10^4^ A W^−1^, and 7.63 × 10^12^ Jones, when the thickness decreases from 63 L to 18 L. The *R*_ex_, *R*_in_, and D* of α-6T increase from 6.79 × 10^2^ A W^−1^, 1.60 × 10^4^ A W^−1^, and 3.63 × 10^12^ Jones to 1.29 × 10^4^ A W^−1^, 6.08 × 10^5^ A W^−1^, and 6.90 × 10^13^ Jones, when the thickness decreases from 8 L to 4 L. All of the data (Fig. 4f–i) show obvious enhancement of photoresponse in the case of the organic crystal at the 2D limit on SiO_2_/Si.

### 2D crystalline structure and optical properties

**Fig. 5:**
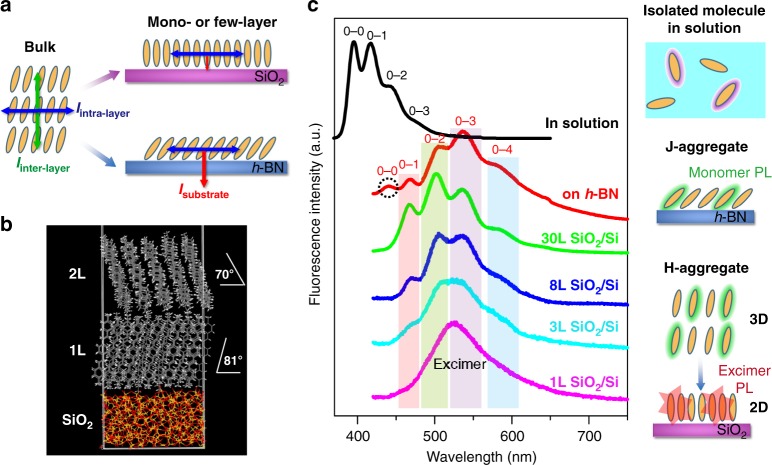
Crystalline structure and optical properties. **a** Schematic illustration of 2D crystals on different substrates (SiO_2_ or *h*-BN). Unlike their bulk counterparts, molecular packing in the first few molecular layers is governed by the competition between the intra-layer interaction (*I*_intra-layer_) and molecule–substrate interaction (*I*_substrate_). On *h*-BN, strong *I*_substrate_ leads to a tilted configuration; while on SiO_2_, weak *I*_substrate_ leads to an approximately freestanding configuration. **b** The optimized model of p-MSB molecular layers on amorphous SiO_2_. **c** PL spectra of p-MSB in solution, p-MSB crystals on *h*-BN, and SiO_2_/Si. The right insets are schematic diagrams for the fluorescence mechanism. Excimer-like emission is obtained when the p-MSB molecules are tightly packed in a 2D crystal on SiO_2_

The enhanced photoresponse is attributed to the unique crystalline structure at the 2D limit. As we know, the organic crystals are bonded via van-der-Waals force, which is much weaker than the covalent bonds in 2DACs. Thus, the crystalline structure as well as the properties of an organic crystal can be easily tuned in a wide range^14,46^ compared with the atomic crystals, especially at the 2D limit. In an organic crystal, three interactions act on a molecule (Fig. 5a), which are molecule–substrate interaction (*I*_substrate_), intra-layer (*I*_intra-layer_), and inter-layer (*I*_inter-layer_) van-der-Walls interactions between adjacent molecules. Owing to the reduced *I*_inter-layer_ compared with the bulk counterparts, molecular packing in few molecular layers is greatly influenced by the competition between *I*_intra-layer_ and *I*_substrate_. For instance, the pentacene molecules in the first layer adopt a completely tilt-free herringbone motif on amorphous SiO_2_, giving rise to high mobility in thin-film transistors^47,48^, in stark contrast to the face-on or largely tilted configuration on hexagonal boron nitride (*h*-BN), which leads to a low-conducting or non-conducting interfacial layer^6^. On the substrates like graphene or *h*-BN, the strong *I*_substrate_ dominates molecular packing of the 2D crystal; thus, the molecules adopt face-on or a largely tilted configuration, as demonstrated previously^6,9^. Control experiment on *h*-BN (Supplementary Fig. 31) shows good agreement with the literature, that a decrease of the layer thickness (Supplementary Table 2), as well as an expansion along *b* axes (Supplementary Figs. 32, 33) are observed when the crystal thickness decreases to one to several molecular layers. On SiO_2_/Si, the *I*_inter-layer_ decreases as the crystal dimension gradually transforms from bulk toward 2D. Owing to the weak *I*_substrate_^47,48^, the *I*_intra-layer_ dominates the molecular packing at the 2D limit, similar with pentacene molecules on SiO_2_^47,48^, leading to a decrease of the tilt angle (from 30° for bulk to 9° for an approximately vertically standing monolayer), shrink along the *b* axes, and increase of the layer thickness, in agreement with the AFM and DFT calculation results (Fig. 5b).

The difference in molecular packing leads to different 2D limit effects on the optical properties. Owing to the molecular aggregation, characteristic PL peaks of the p-MSB crystals bathochromically shift to around 466 nm (0–1 band), 502 nm (0–2 band), 536 nm (0–3 band), and 584 nm (0–4 band), compared with isolated molecules in solution. In the form of solid films and crystals, the PL spectroscopy is strongly dependent upon the molecular packing^49^. In the case of the p-MSB crystals on *h*-BN, the 0–0 band at 439 nm indicates the existence of the J-aggregate where the molecules are arranged in a head-to-tail direction, in agreement with face-on or a largely tilted configuration. In the case of the p-MSB crystals on SiO_2_/Si, the 0–0 band is strongly suppressed. The H-aggregate is expected where the molecules are aligned parallel (face-to-face) to each other with strong intermolecular interaction, since the 0–0 band is forbidden due to Davidov splitting associated with the H-aggregate^50^. Along with decreasing thickness from 20 L to 1 L, p-MSB characteristic peaks gradually disappear, while a new red-shifted, broad, and unstructured emission bands appear at around 426 nm, indicating the formation of excimer-like states. The excimer is an excited-state species, where the frontier orbital electron density is overlapped and shared equally between neighboring chromophores^50^. To form an excimer, strong electronic coupling as well as tightly face-to-face H-aggregate molecular packing is required^49,50^. The nearly pure excimer emission reveals the highly compact structure of the 2D p-MSB crystal, and reveals that owing to the strong coupling between adjacent molecules, new energy bands form and the p-MSB molecules behave more like an aggregation rather than isolated molecules at the 2D limit on SiO_2_/Si.

### Photoresponse enhancement mechanism at the 2D limit

**Fig. 6:**
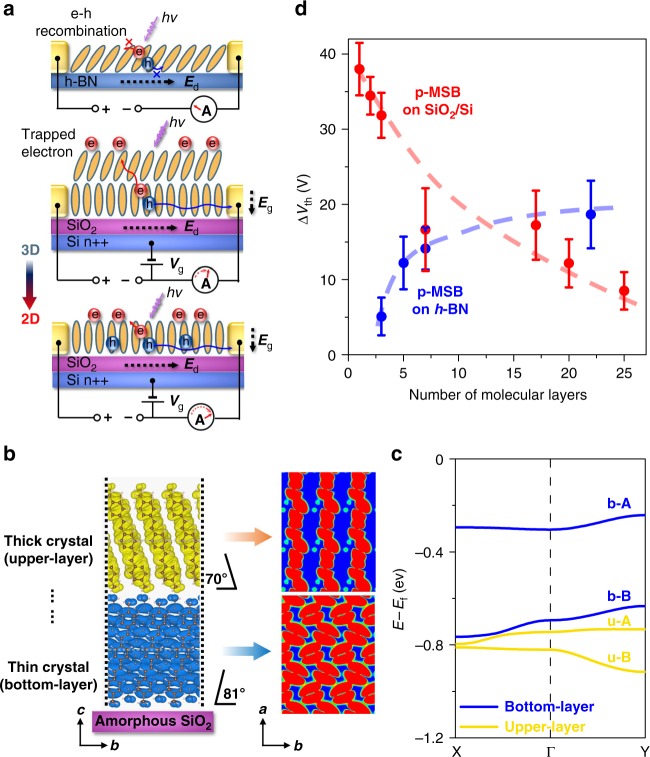
Enhancement mechanism of the photoelectrical response. **a** Schematic illustration of photoresponse mechanism on different substrates (SiO_2_ or h-BN). **b** Calculated molecular orbitals of the intermolecular bonding states for the upper and bottom p-MSB molecular layers on amorphous SiO_2_ in the *b*–*c* plane (side view) and the *a–b* plane (top views). The white dashed lines in the side view indicate the positions of the slices of the top view. **c** Calculated band structures of the upper and bottom p-MSB molecular layers on amorphous SiO_2_, which includes four states, namely u-A (upper-“anti-bonding”), u-B (upper-“bonding”), b-A (bottom-“anti-bonding”), and b-B (bottom-“bonding”). The “anti-bonding” and “bonding” refer to the intermolecular interactions. **d** Dependence of threshold voltage shift (Δ*V*_th_) on the number of the p-MSB molecular layers under a 14.1 μW cm^−2^ 365-nm light. The error bars in **d** represent the s.e.m. of the Δ*V*_th_ measured from the transfer curves

Similar like a 2D limit effect on PL emission, the difference in crystalline structure, especially the shrink or expansion along the *b* axes, which is the main conducting direction, leads to different 2D limit effects on the electrical properties, as well as the photoelectrical response of the organic crystals on different substrates (Fig. 6a).

On SiO_2_/Si, we speculate three reasons for the photoreponse enhancement. The first is the band-like carrier transport. First-principle calculation of multilayer p-MSB molecules on amorphous SiO_2_ (Fig. 6b) shows that the molecules in the bottom layer are uprightly standing with an immanent tilt angle of 9°, while the molecules in the upper layer are tilted at an angle of 20°. Figure 6c shows the calculated band dispersion for the bottom and the upper layers. The intermolecular antibonding state is higher than the intermolecular bonding states, indicating that hole transport is the main conducting mechanism. The H-aggregated bottom layer has a much shallower antibonding state of the highest-occupied molecular orbital (HOMO) compared with that of the upper layer, which probably allows efficient band-like hole transport to increase hole mobility in 2D crystals (0.158 cm^2^ V^−1^ s^−1^ for 8 L) compared with that in bulk samples (0.053 cm^2^ V^−1^ s^−1^ for 85 L). This result is consistent with the calculated molecular orbitals of the intermolecular bonding states for the upper and the bottom layers (Fig. 6b). In the upper layer, tilted molecular packing leads to localized molecular orbitals. The carriers transport by a hopping mode, which gives rise to the low efficient separation and transfer of photo-generated carriers due to the small localization length^51,52^. Thus, a degraded photoelectrical response is expected for thick or bulk crystals. In the bottom layer, the horizontally overlapped orbitals form a continuous network along *a* and *b* axes, contributing to a band-like transport mode. In this case, longer exciton or charge diffusion length is expected^53,54^, which avoids recombination of the photo-generated carriers and gives rise to the highly efficient carrier transfer in an external electrical field (*E*_d_).

The second is the efficient charge separation. The electron–hole pair created via photon absorption must overcome Coulomb attraction to achieve long-range charge separation. Previous literatures show that the charge carrier and exciton delocalization plays a significant role in ultrafast charge separation^55^, and a small reduction in the intermolecular stacking distance can not only significantly improve the carrier mobility, but also turn the photoexcitation and carrier transport to a band-like mechanism^54^, resulting in a band-like excimer emission of the 2D p-MSB crystal. Such strong electron cloud overlapping between H-aggregated molecules leads to effective enhancement of long-range charge separation in the organic crystal^22,55^. Owing to the more vertically standing p-MSB molecules and the resulting highly compact structure of the 2D crystal on SiO_2_/Si, efficient charge separation takes place, which provides more free charges for increasing the photoconduction and the photo-gating modulation.

The third is the enhanced photo-gating effect. Photo-gating effect is a widely used strategy for improving light response performance of 2D or organic photodetectors, which introduces a light-induced electrical gate modulation for increasing the photocurrent in the device channel^56^. Under illumination, electron–hole pairs are generated and subsequently dissociated into electrons and holes under the bias and back-gate electrical field (*E*_g_). The holes are transferred in the p-type channel to the electrodes, while some photo-generated electrons are captured on the crystal surface or in itself^22,54^ with a trap density of 4.73 × 10^12^ cm^−2^ (43.1 μW cm^−2^ 254-nm light) and a lifetime up to 260 ms (Supplementary Note 7, Supplementary Fig. 34). This long lifetime might be related to the weak binding between electron–hole pairs^54^, and it also indicates an efficient electron-trapping capability of the 2D p-MSB crystal; otherwise, these electrons would be swept out by *E*_d_^54^. The confinement of holes in a narrow region leads to a spatial separation between the transporting holes and the trapped electrons, which reduces their recombination^54^. The trapped electrons produce an additional electric field-like local gate voltage, which positively shifts the *V*_th_ (Fig. 6d, Supplementary Fig. 35) and introduces more holes in the p-type channel, leading to an increase of the photoresponsivity^22^. With decreasing layer numbers on SiO_2_/Si, the tightly packed structure gives rise to higher charge separation efficiency^54^, providing more electrons for photo-gating modulation. This, as well as a stronger doping effect of the trapped electrons in the thinner crystal^56^, results in a remarkably enhanced photo-gating effect. Therefore, a larger *V*_th_ shift (Δ*V*_th_) is observed with decreasing thickness (Fig. 6d), leading to a larger photoresponse in the 2D p-MSB crystal on SiO_2_/Si.

On the *h*-BN, the 2D limit effect is distinct. Previous literatures demonstrate that J-aggregate tilted molecular packing results in localization of the density of states and a distinct band structure from bulk crystals, which hampers intermolecular charge transfer, giving rise to a non-conducting or low-conducting interfacial layer^6,9^. The mobilities are about 0.00229 and 0.00524 cm^2^ V^−1^ s^−1^ for 3 L and 5 L p-MSB samples, respectively, much lower than that of bulk samples (0.053 cm^2^ V^−1^ s^−1^ for 85 L). The carriers or excitons can only travel a small distance in the 2D crystal, i.e., the localization length of a hole is only ∼1 nm in monolayer pentacene on *h*-BN^6^. Thus, under light irradiation, the highly tilted structure not only reduces the carrier transport, but also decreases the separation of photo-generated carriers and increases the recombination of holes and the trapped electrons^54^, leading to lower photo-generated carrier density and weaker photo-gating modulation with decreasing the thickness (Fig. 6d). As a result, the *R*_in_ decreases from 8.23 × 10^5^ to 1.47 × 10^5^ A W^−1^ when the thickness decreases from 8 L to 3 L, as shown in the control experiments (Fig. 4g). Therefore, lower photoelectrical response is expected at the 2D limit on *h*-BN.

Moreover, without any treatment of the SiO_2_/Si, the mobilities and *R*_in_ of the monolayer and bilayer samples are 0.004–0.007 cm^2 ^V^−1^ s^−1^, ~2.84 × 10^4^ A W^−1^ (254-nm light), and 5.35~8.56 × 10^4^ A W^−1^ (365-nm light), respectively, due to the undesired roughness, defects, and dangling bonds on the surface. The performance can be improved by modifying the substrate using a clean Van-der-Walls surface or self-assembly molecular layers to reduce the amount of roughness and charge impurity traps. As a result, on the high-quality surface of the two bottom layers, the p-MSB crystals achieve the highest *R*_in_ at 3 L.

## Discussion

In this article, we develop a simple method to grow large-sized 2D p-MSB single crystals under thermodynamically controlled conditions. This method is not only limited to p-MSB, but also valuable for growth of high-quality 2DOCs made of other molecules. More interestingly, the organic crystal on SiO_2_/Si exhibits distinct 2D dimension effect from that of 2DACs or organic crystals on *h*-BN. When decreasing the thickness down to several molecular layers, the *R*_in_ increases by 2–3 orders compared with that of bulk samples. The p-MSB molecules become more vertically stacked at the 2D limit on SiO_2_/Si, owing to the weak *I*_substrate_. As a result, the molecular orbitals overlap horizontally, and the corresponding band structure changes, leading to a band-like excimer emission and an increase of the photoelectrical response. To the best of our knowledge, it is the first report that the photoelectrical response of an organic crystal is enhanced at the 2D limit. This work extends the fundamental understanding of the principles for 2D epitaxial growth of organic crystals and the 2D dimension effect on their photoelectrical properties, opening up a promising avenue toward high-performance photoelectrical devices based on 2DOCs.

## Methods

### Growth of 2D p-MSB crystal

A SiO_2_/Si substrate was washed by ultrasonication in acetone and deionized, respectively, and then was blown dry using a N_2_ gun. 2D p-MSB crystals were prepared by a drop-casting method. In total, 2.0 mg of p-MSB (98%, TCI Chemicals) powder was added in 10 mL of toluene, and was subjected in an ultrasound bath at 60 °C for 30 min to achieve the complete dissolution. The oversaturated solution was then slowly cooled down to room temperature without agitation to prepare the seed crystals. After being heated in an oven at 80 °C for 5 min, a drop of solution (20 μL) containing seed crystals was poured over the SiO_2_/Si substrate placed inside a covered Petri dish under ambient atmosphere, allowing the solvent to evaporate slowly. To maintain the temperature, the covered Petri dish was put on a hot plate. After the growth, the as-grown crystals were annealed under N_2_ atmosphere at 80 °C for 30 min to ensure the complete removal of the solvent.

### Characterization

The samples were characterized by a polarized optical microscope (Leica DM2500P), SEM (Zeiss Ultra 55, acceleration voltage: 5 kV), TEM (FEI Tecnai G2 F20 S-Twin, acceleration voltage: 200 kV), XRD (Rigaku D/Max 2400, CuKα radiation, 40 kV, 100 mA, *λ* = 1.5406 Å) and AFM (Multimode 8, Bruker, tapping mode). For TEM measurement, a drop of p-MSB oversaturated solution was poured onto a copper grid placed inside a covered Petri dish. The 2D crystals were grown on the surface of copper grid. High-resolution AFM (Asylum Cypher ES, Oxford Instruments) measurement was performed under ambient conditions. The UV-vis absorption spectrum was recorded by a Lambda 750 spectrometer. Synchrotron radiation GIXRD was performed at BL14B beamline, Shanghai synchrotron radiation facility with a wavelength of 1.24 Å. GIXRD data were acquired by using a MarCCD with a distance of ~284 mm from the samples^57^. The grazing incidence angle was fixed at 0.1° or 0.5° and the exposure time was set to 30 s.

### Device fabrication and measurement

2D single crystals of p-MSB, perylene, and α-6T were directly prepared on SiO_2_/Si. Drain and source electrodes (Au with 50-nm thickness) were deposited on the crystal by thermal evaporation (Nexdep, Angstrom Engineering Inc.) using a shadow mask. The electrical measurement was carried out by a probe station (EVERBING, PE-4) and a semiconductor analyzer (Keysight B1500A). Monochromatic light (wavelength: 254, 365, 405, 410, 420, and 500 nm) was applied to the samples through an optical fiber. The power intensity of the light was measured by a standard photodiode power sensor (S120VC, Thorlabs). All measurements were performed at room temperature in the ambient atmosphere.

### First-principles calculations

First-principles calculation was performed using DFT from Vienna ab initio simulation package with a plane-wave basis^58^. Local density approximation (LDA) was used for the exchange and correlation functional^59^, and van-der-Waals (vdW) corrections to the density functional in the Grimme implementation^60^ were also included. The core electrons were represented by the projector-augmented-wave (PAW) potential^61^. The kinetic energy cutoff was set above 400 eV. The geometrical structure of molecule layers was obtained based on an optimized structure on amorphous SiO_2_ (Fig. 5b). The structure optimization process of the crystal on amorphous SiO_2_ was performed by DFTB^62^ with force convergence criteria at 0.05 eV/Å. The surface energy is defined as *E*_surface_ = *E*_slab_−*E*_bulk_/2*S*, where *E*_slab_ is the total energy of the slab with vacuum, *E*_bulk_ is energy of bulk molecule crystal without vacuum, and *S* is the surface area.

## Supplementary Information

Supplementary Information

Description of Additional Supplementary Files

Supplementary Movie 1

Supplementary Movie 2

Supplementary Movie 3

## Data Availability

The data that support the findings of this study are available from the corresponding author upon request.
