# Supplementary material for: Enhanced photoelectrical response of thermodynamically epitaxial organic crystals at the two-dimensional limit
Source: Nat Commun. 2019 Feb 14;10:756. doi: 10.1038/s41467-019-08573-8 (PMC6375977; doi:10.1038/s41467-019-08573-8)
Supplement: Supplementary file 1 — Supplementary Information [file 41467_2019_8573_MOESM1_ESM.pdf]

Supplementary Information for

"Enhanced Photoelectrical Response of Thermodynamically Epitaxial Organic Crystals at the Two-Dimensional Limit"

Cao et al.

Supplementary Figures

Supplementary Figure 1

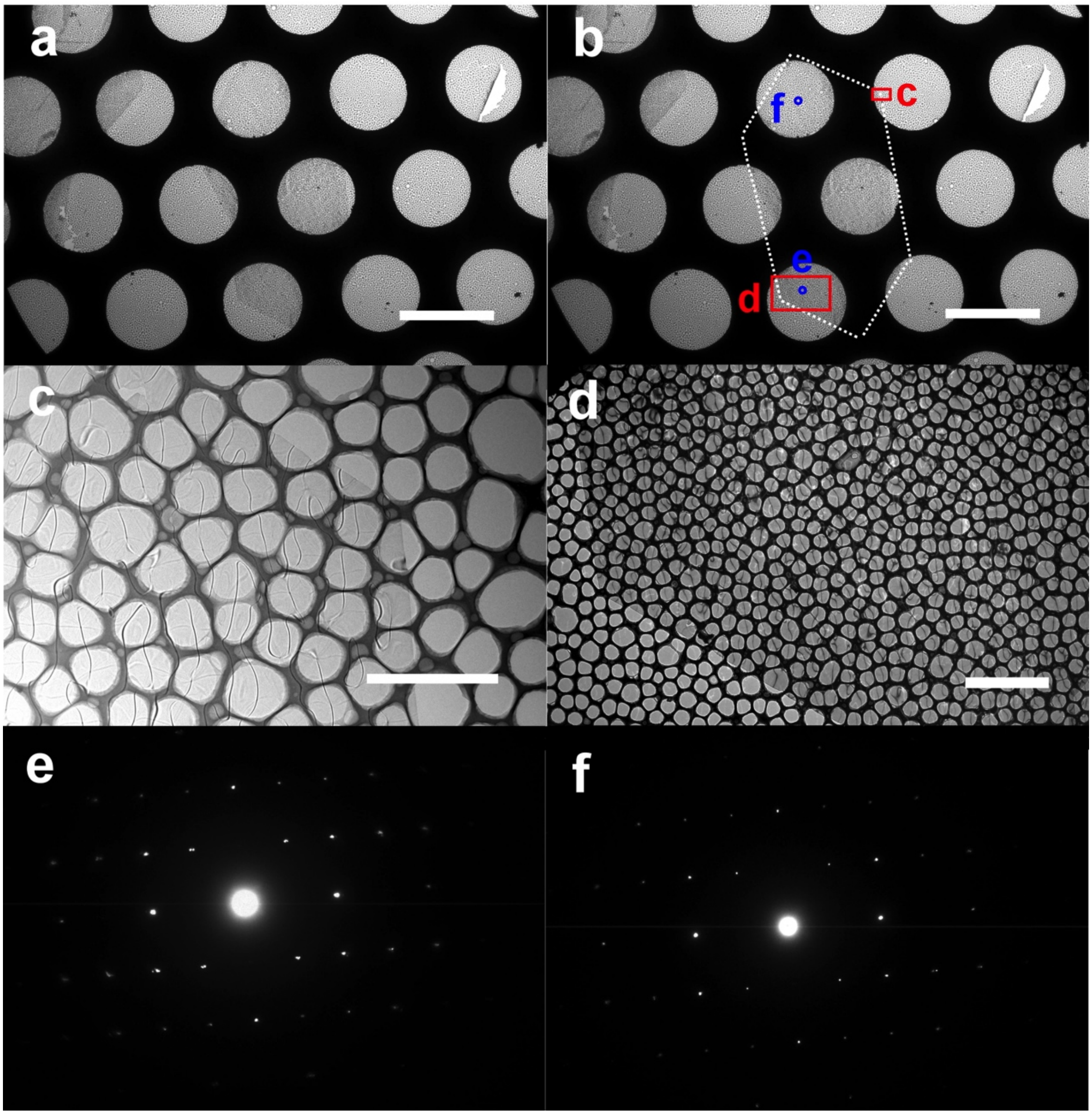

Supplementary Figure 1. TEM images and SAED patterns of a 2D p-MSB crystal. **c** and **d** are collected from areas marked by red flames in **b**. **e** and **f** are collected from the areas marked by blue circles in **b**. The scale bars are 100 μm in **a** and **b**, 5 μm in **c**, and 10 μm in **d**.

Supplementary Figure 2

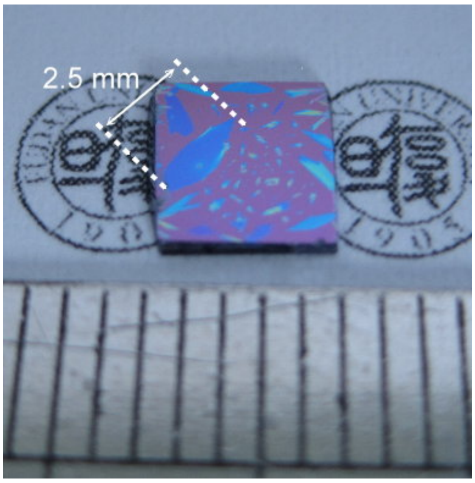

Supplementary Figure 2. Photograph of a 2D p-MSB crystal with size up to 2.5 mm.

Supplementary Figure 3

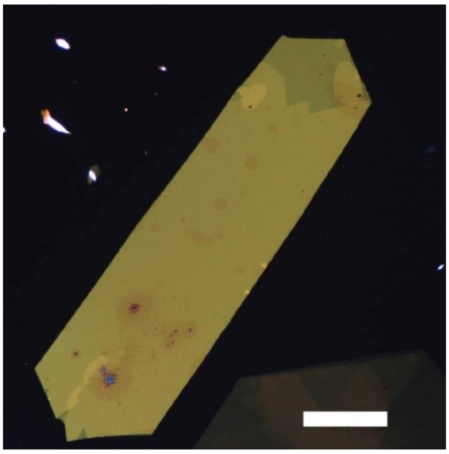

Supplementary Figure 3. Optical microscope image. Cross-polarized optical microscope image of a p-MSB 2D crystal. The scale bar is 200 μm.

Supplementary Figure 4

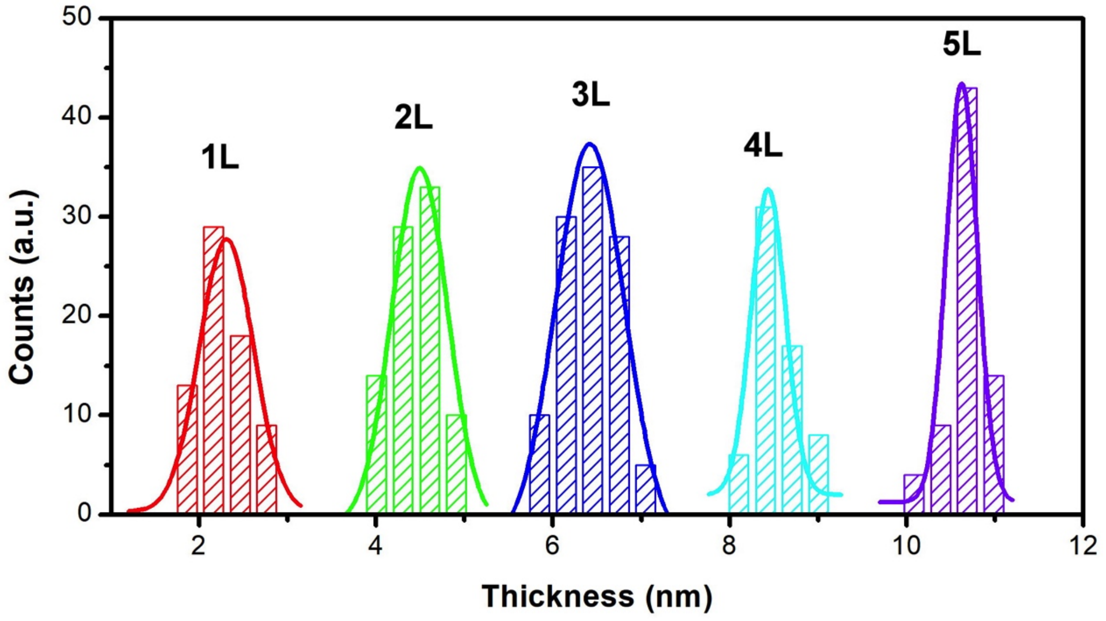

Supplementary Figure 4. The thickness distribution. The thickness distribution (measured by AFM) of the 2D p-MSB crystals with different layer numbers (1L to 5L).

Supplementary Figure 5

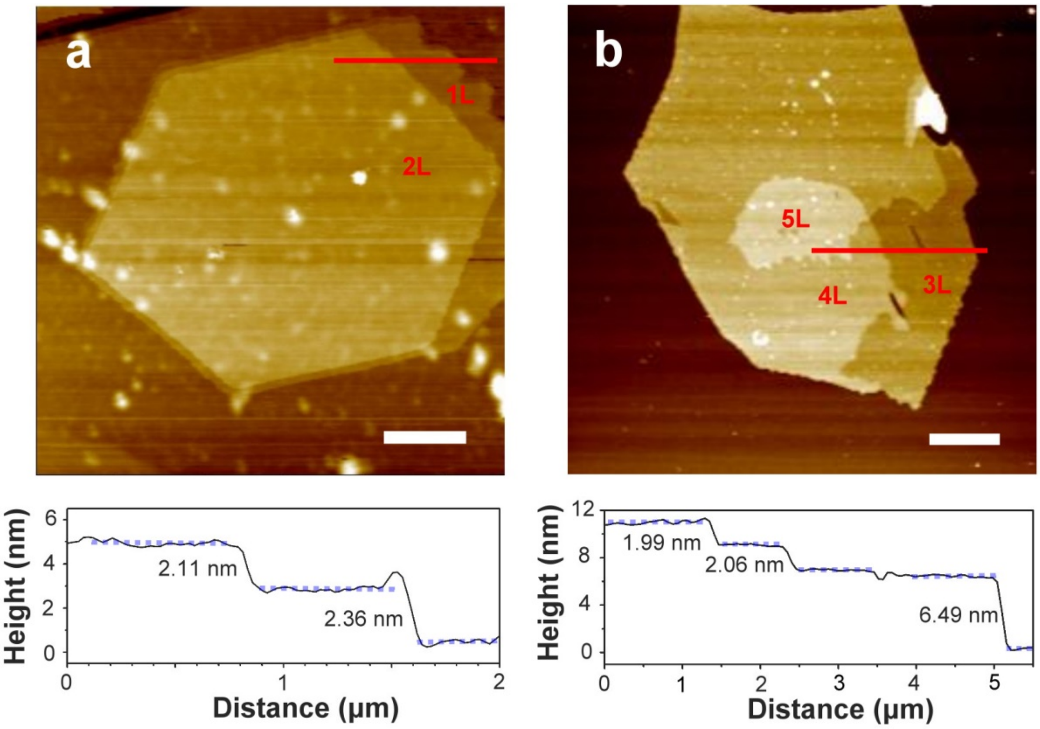

Supplementary Figure 5. AFM images of 2D p-MSB crystals. AFM images of 2D p-MSB crystals with different thickness, which clearly show that the bottom layers are thicker compared with the upper layers. The lower insets are the height profiles along the red lines in the AFM images. The scale bars are 1 μm in **a**, and 2 μm in **b**.

Supplementary Figure 6

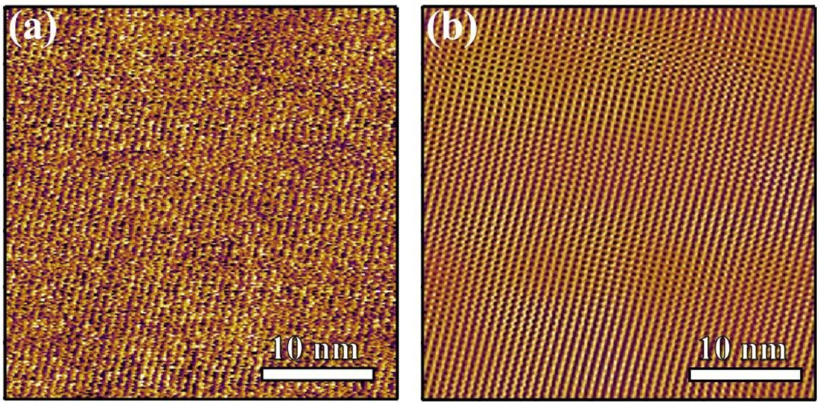

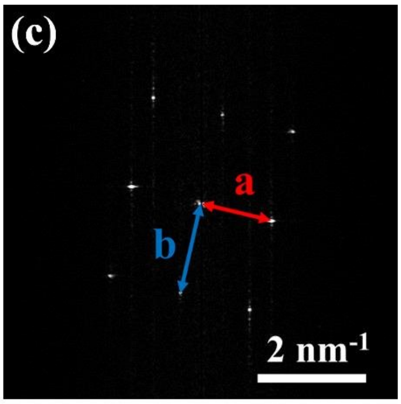

(d)

Thickness	monolayer (2 nm)
a/nm^{-1}	1.326 ± 0.008
b/nm^{-1}	1.681 ± 0.007
a/Å	7.54 ± 0.05
b/Å	5.95 ± 0.02

Supplementary Figure 6. High-resolution AFM of a mono-layer p-MSB crystal. **a,** High-resolution AFM image (lateral trace) and **b,** the corresponding image processed by fast Fourier transform (FFT) filtering technique. **c,** FFT pattern of the high-resolution AFM image. **d,** Lattice parameters of the mono-layer p-MSB crystal calculated from the FFT pattern. The scale bars are 10 μm in **a** and **b**, 2 nm^{-1} in **c**.

Supplementary Figure 7

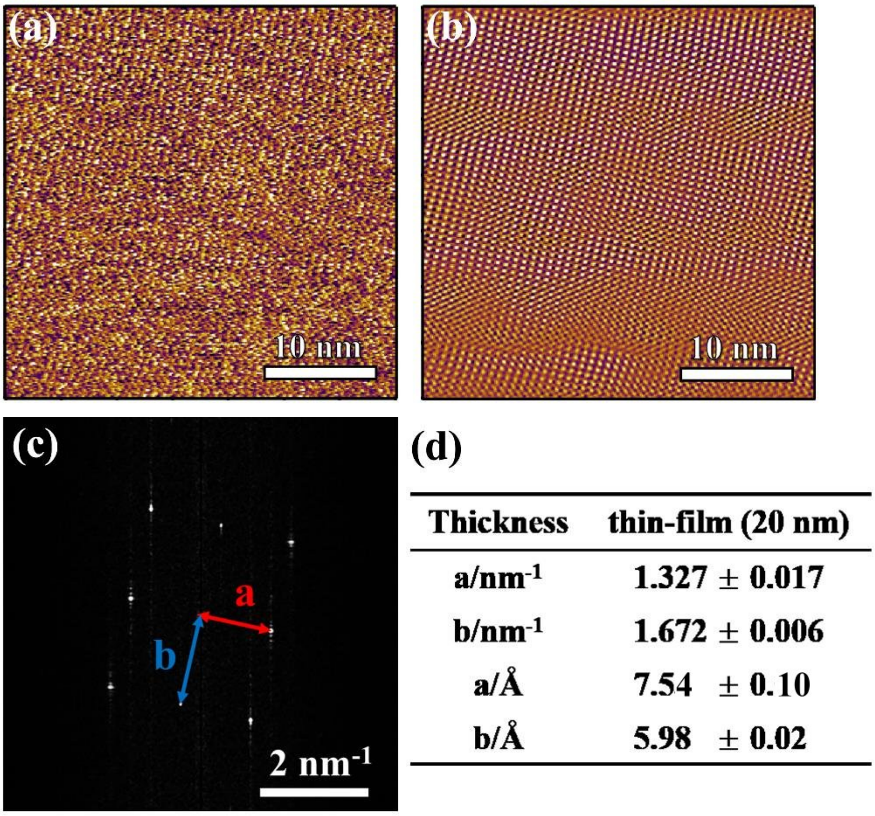

Thickness	thin-film (20 nm)
a/nm^{-1}	1.327 ± 0.017
b/nm^{-1}	1.672 ± 0.006
a/Å	7.54 ± 0.10
b/Å	5.98 ± 0.02

Supplementary Figure 7. High-resolution AFM of a thin (20 nm) p-MSB crystal. **a,** High-resolution AFM image (lateral trace) and **b,** the corresponding image processed by fast FFT filtering technique. **c,** FFT pattern of the high-resolution AFM image. **d,** Lattice parameters of the thin p-MSB crystal calculated from the FFT pattern. The scale bars are 10 μm in **a** and **b**, 2 nm^{-1} in **c**.

Supplementary Figure 8

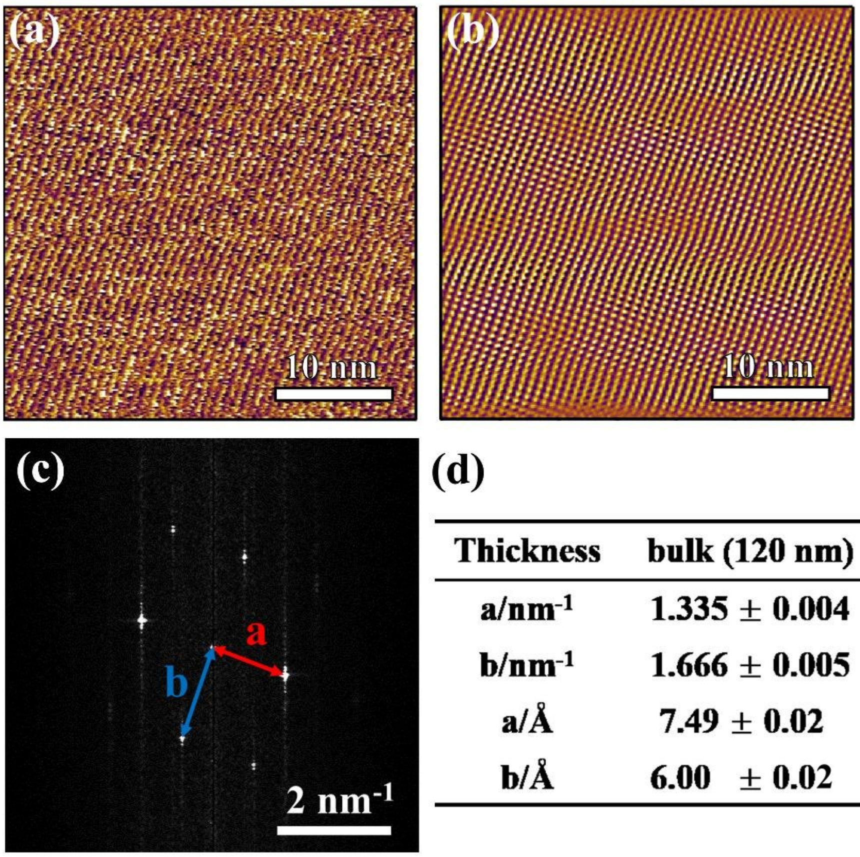

Thickness	bulk (120 nm)
a/nm^{-1}	1.335 ± 0.004
b/nm^{-1}	1.666 ± 0.005
a/Å	7.49 ± 0.02
b/Å	6.00 ± 0.02

Supplementary Figure 8. High-resolution AFM of a bulk (120 nm) p-MSB crystal. **a,** High-resolution AFM image (lateral trace) and **b,** the corresponding image processed by fast FFT filtering technique. **c,** FFT pattern of the high-resolution AFM image. **d,** Lattice parameters of the thin p-MSB crystal calculated from the FFT pattern. The scale bars are 10 μm in **a** and **b**, 2 nm^{-1} in **c**.

Supplementary Figure 9

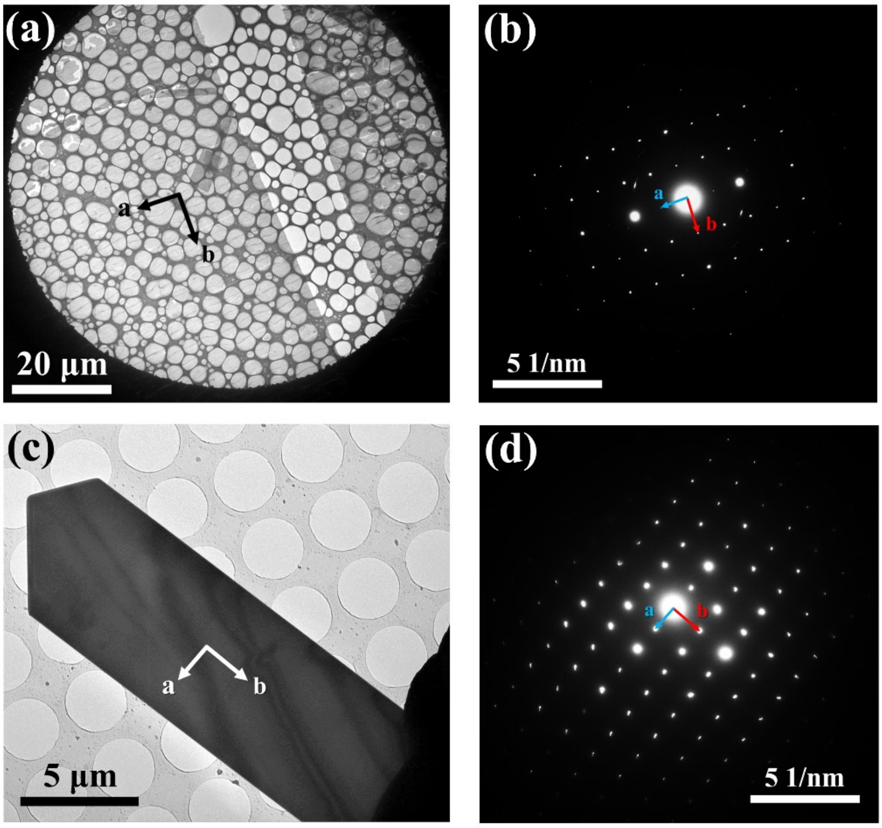

(e)

	Thin-film (~20 nm)	Bulk (~160 nm)
b/a	0.774	0.782

Supplementary Figure 9. TEM of the p-MSB crystals with different thickness. **a-d,** TEM and SAED analysis of **a,b,** a thin and **c,d,** a bulk crystal of p-MSB, respectively. **e,** The b/a ratio of the thin-film and bulk crystal of p-MSB calculated from the SAED image. Compared with the bulk crystal, the ratio of the lattice constants b/a of the thin crystal is smaller, indicating a more compact π-stacking along the b direction. The scale bars are 20 μm in **a**, and 5 μm in **c**, and 5 nm^{-1} in **b** and **d**.

Supplementary Figure 10

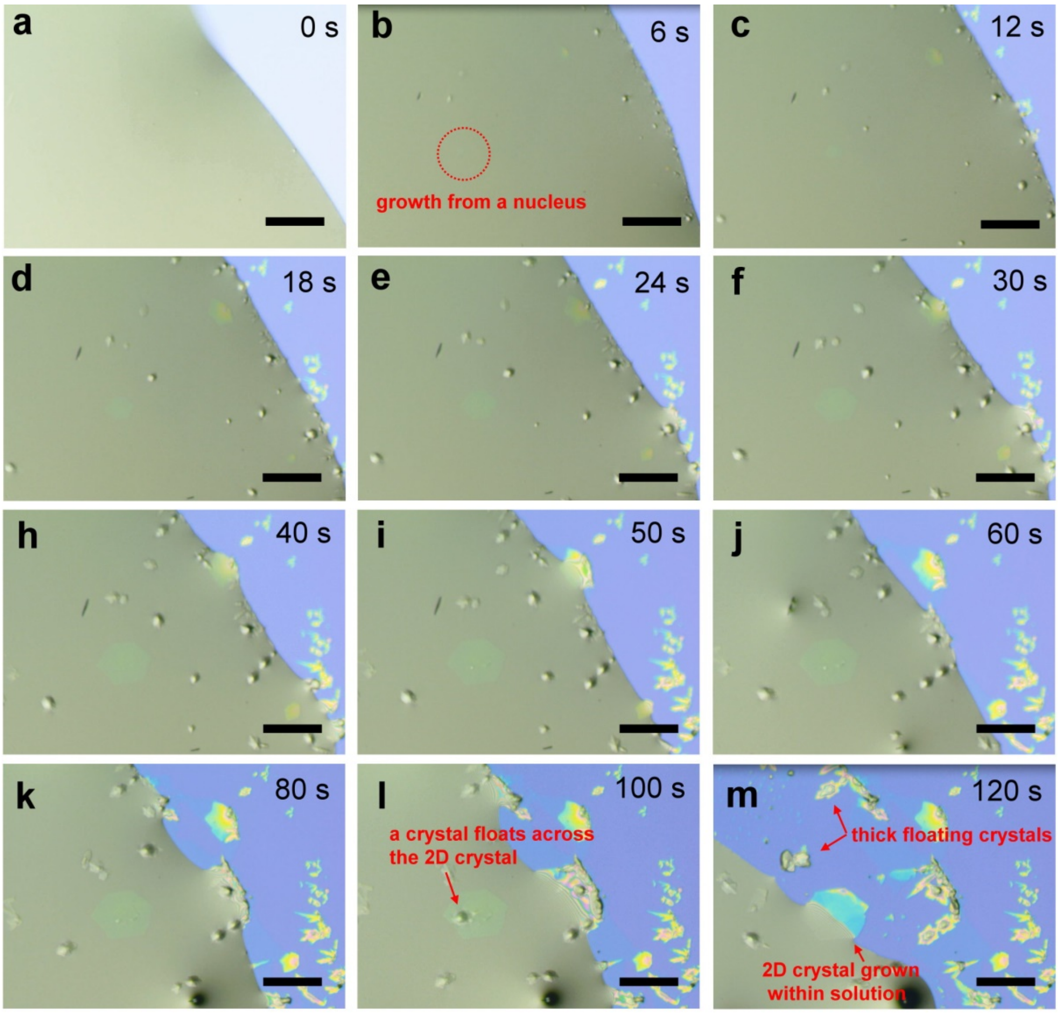

Supplementary Figure 10. In-situ observation of the crystal growth. **a-m,** The images were captured at 0 s, 6 s, 12 s, 18 s, 24 s, 30 s, 40 s, 50 s, 60 s, 80 s, 100 s and 120 s, respectively, after the solution was dropped on SiO_2/Si. It is clear that the growth of 2D p-MSB crystal takes place on the SiO_2/Si surface inside the solution (i.e., it can be proved by **l**, that a crystal floats across the 2D crystal). The video was captured under microscope illumination and the growth took place in an uncovered Petri dish, thus the solvent was evaporated much faster than the actual growth process. Due to the surface nucleation as a result of the fast solvent evaporation, some thick crystals grew on the solution surface. These floating crystals could be avoided by decreasing the evaporation rate in a covered Petri dish. The scale bars are 200 μm.

Supplementary Figure 11

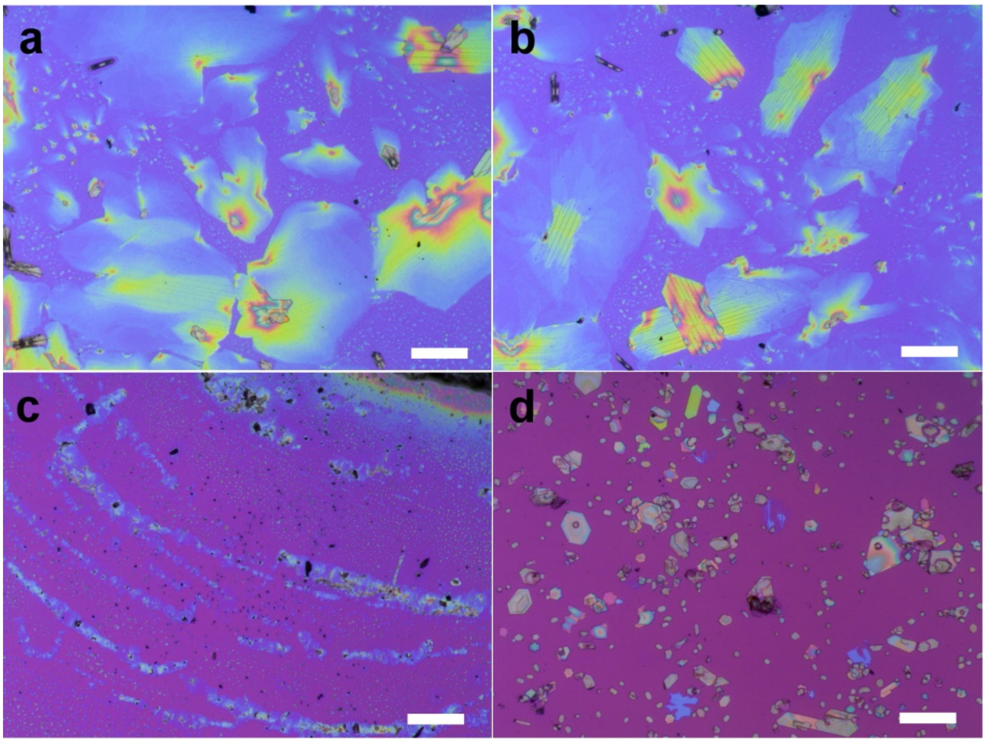

Supplementary Figure 11. The p-MSB samples produced in different conditions. **a, b,** The p-MSB samples produced by quickly evaporating the toluene solvent at 373 K in an uncovered Petri dish. **c,** The samples produced by quickly drying the toluene solvent using nitrogen gas. **d,** The p-MSB samples produced by using an oversaturated toluene solution with a large amount of seed crystals (1 mg mL^{-1}). The scale bars are 200 μm.

Supplementary Figure 12

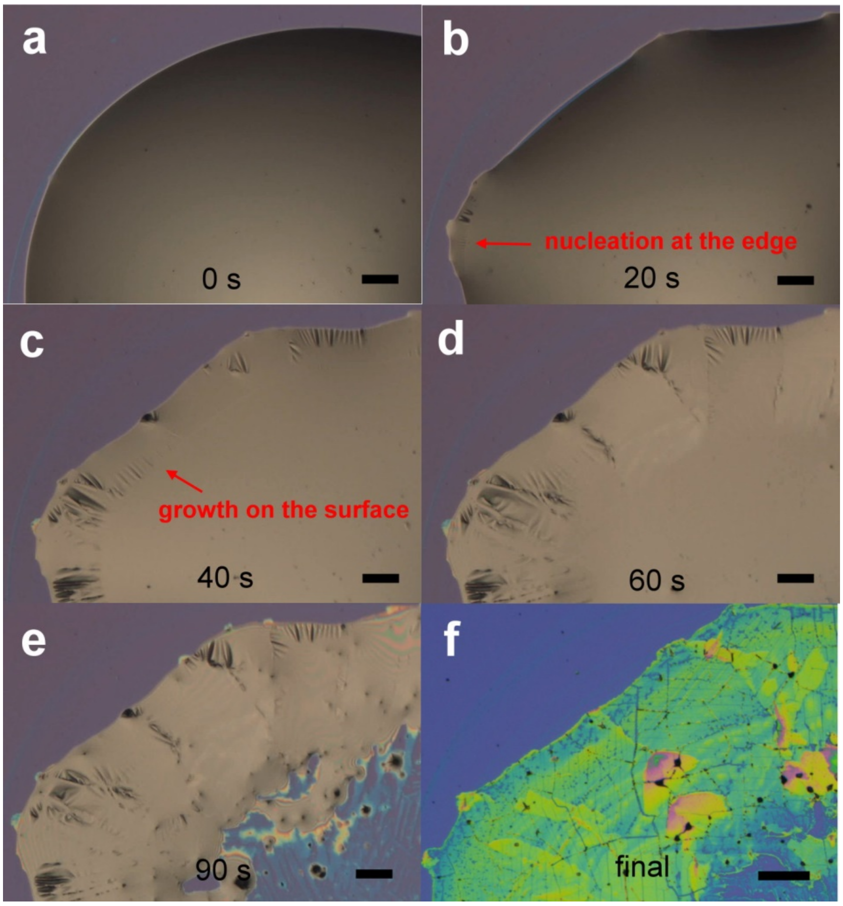

Supplementary Figure 12. In-situ observation of the growth of perylene film. **a-e,** The images were captured at 0 s, 20 s, 40 s, 60 s, 90 s, respectively, after the solution was dropped on SiO_2/Si. **f.** The image was captured after the solution was dried. The growth takes place at the solution (DMP)/air interface under optical microscope observation. The scale bars are 200 μm.

Supplementary Figure 13

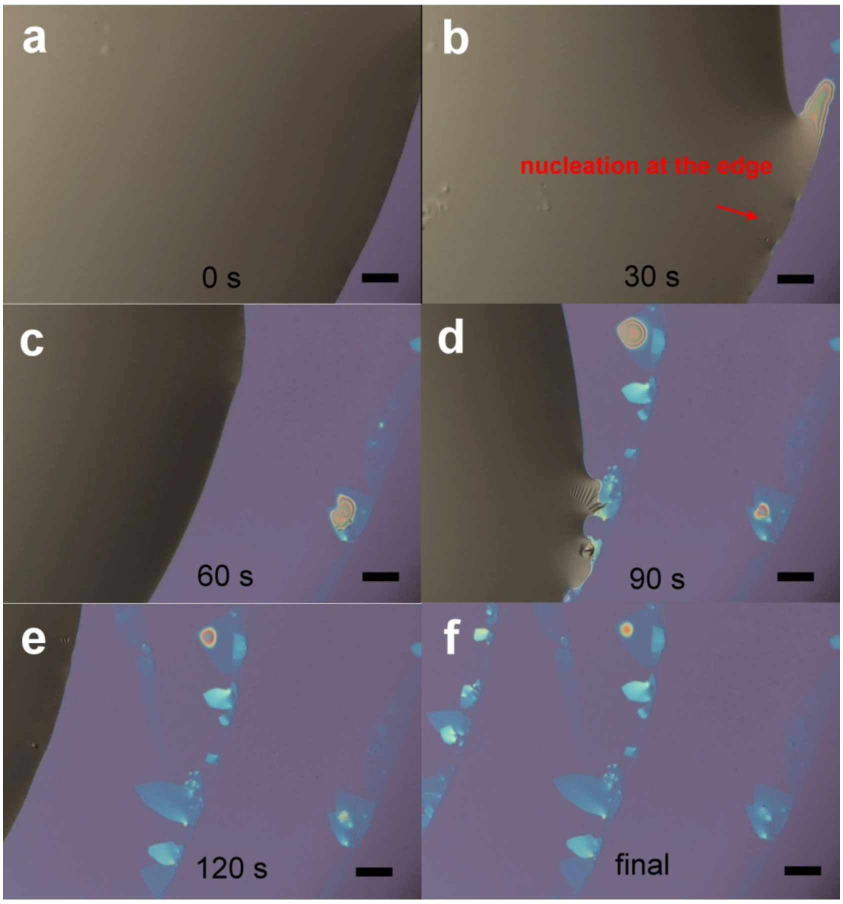

Supplementary Figure 13. In-situ observation of the growth of p-MSB crystals. **a-e,** The images were captured at 0 s, 30 s, 60 s, 90 s, 120 s, respectively, after the solution was dropped on SiO_2/Si. **f.** The image was captured after the solution was dried. The growth takes place at the droplet edge (DMSO) under optical microscope observation. The scale bars are 200 μm.

Supplementary Figure 14

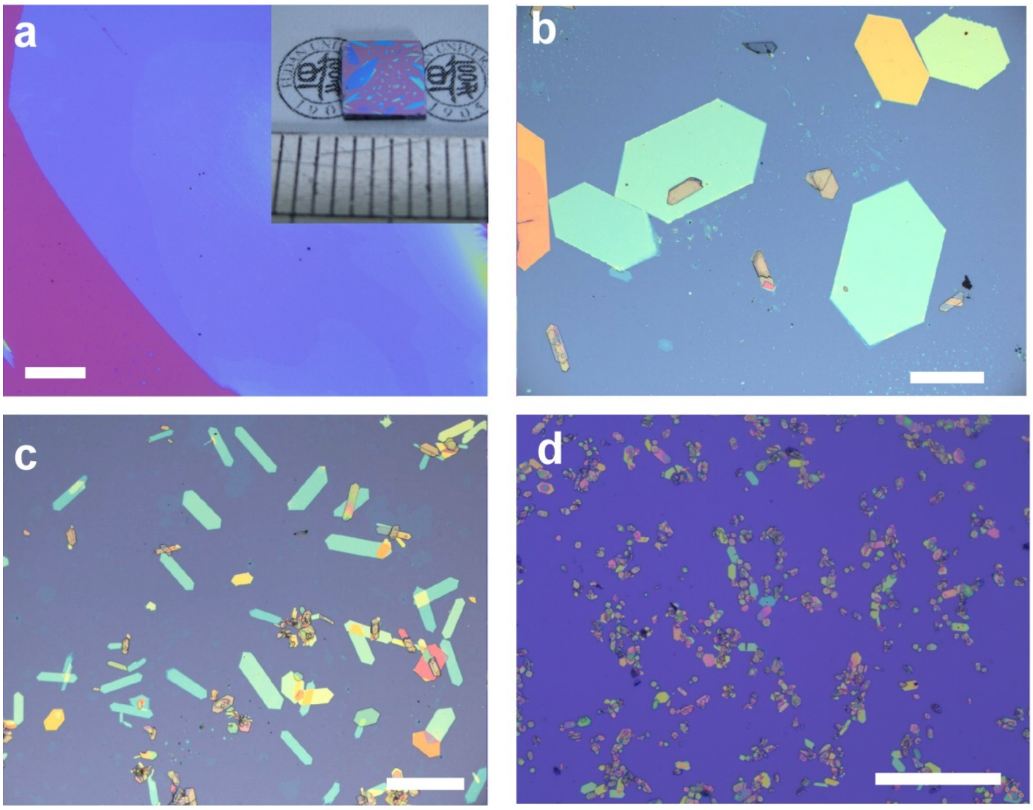

Supplementary Figure 14. The samples produced at different temperatures. **a,** The sample was produced by using hot solution on a hot plate (373 K). The inset is a low-magnified photograph. **b,** The sample was produced by using hot solution in ambient condition. **c,** The sample was produced by using room temperature solution in ambient condition. **d,** The sample was produced by using room temperature solution in ice bath. The scale bars are 200 μm.

Supplementary Figure 15

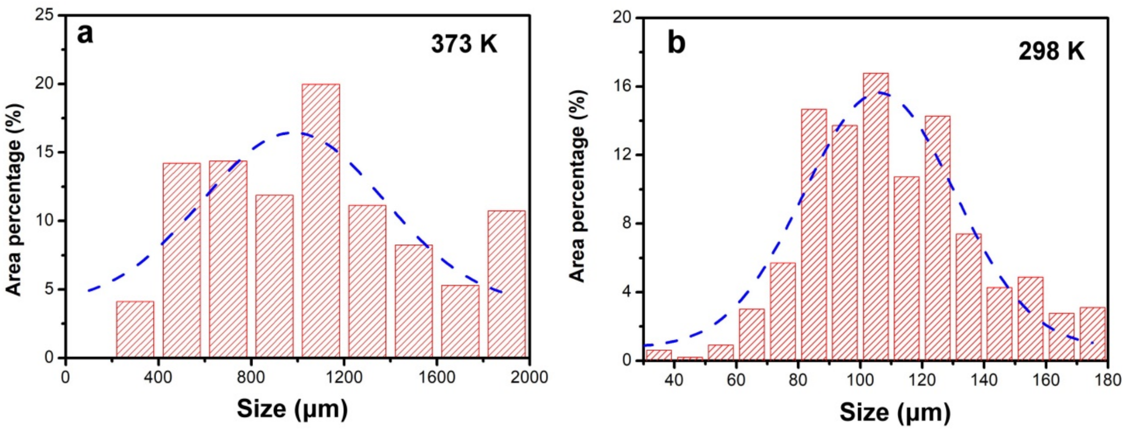

Supplementary Figure 15. The crystal size distribution. **a,** The average size is about 1 mm for the sample produced at 373 K, and **b,** it is about 110 μm for the sample produced at 298 K, indicating that the growth of large-sized 2D p-MSB crystal takes place at higher temperature.

Supplementary Figure 16

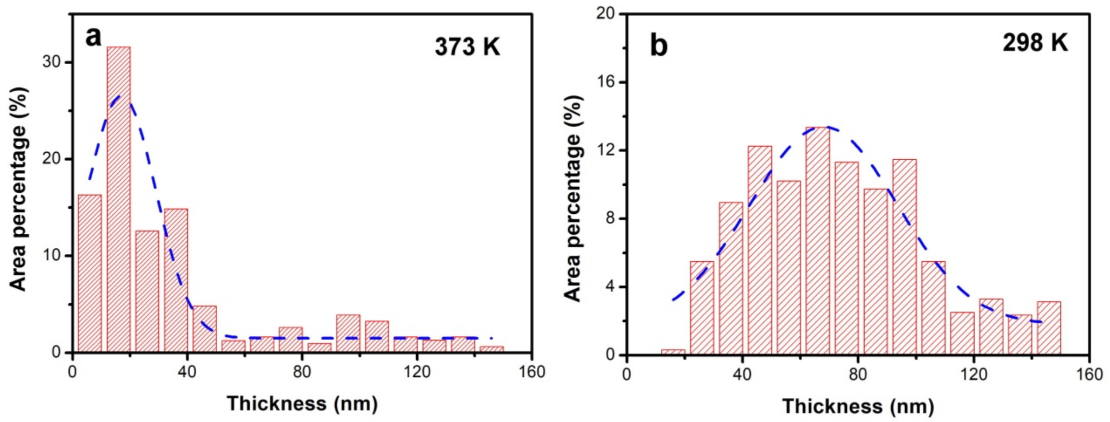

Supplementary Figure 16. The thickness distribution. **a,** The average thickness is about 17 nm for the sample produced at 373 K, and **b,** it is about 65 nm for the sample produced at 298 K, indicating that the growth of thin p-MSB crystal takes place at higher temperature.

Supplementary Figure 17

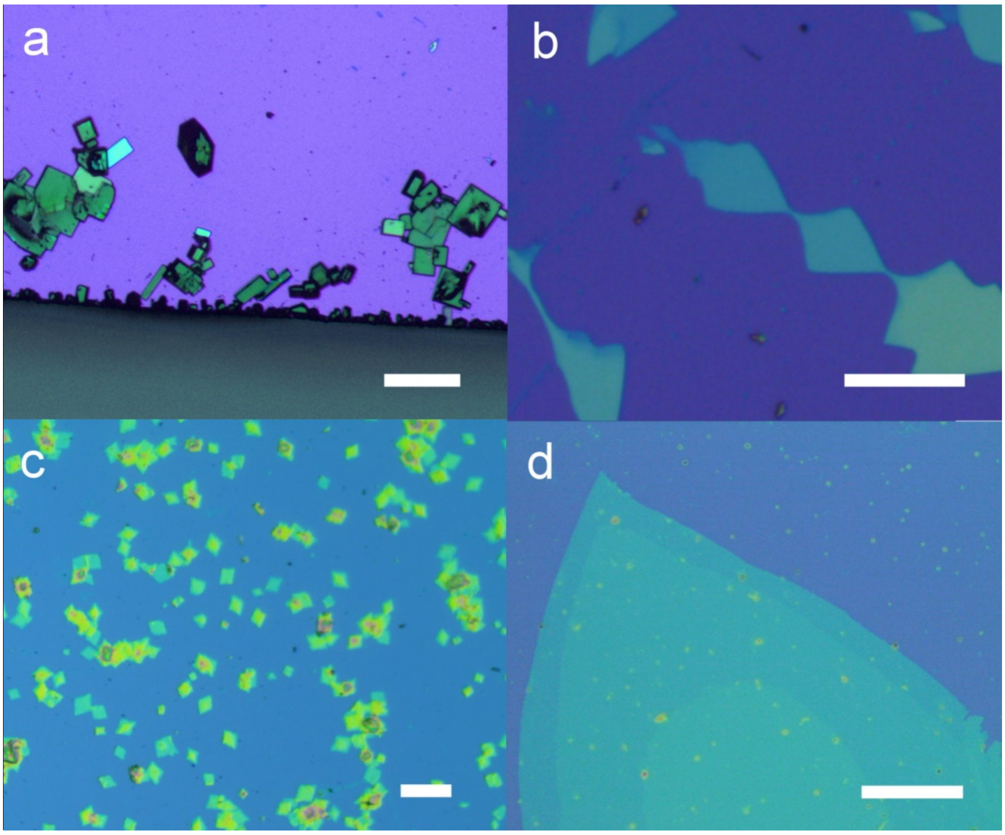

Supplementary Figure 17. The perylene or sexithiophene crystals growth. The crystals are grown by the seed-epitaxial drop-casting method. The perylene crystals are produced at room temperature in **a** and at 353 K in **b**. The sexithiophene crystals are produced at room temperature in **c** and at 353 K in **d**. The scale bars are 100 μm.

Supplementary Figure 18

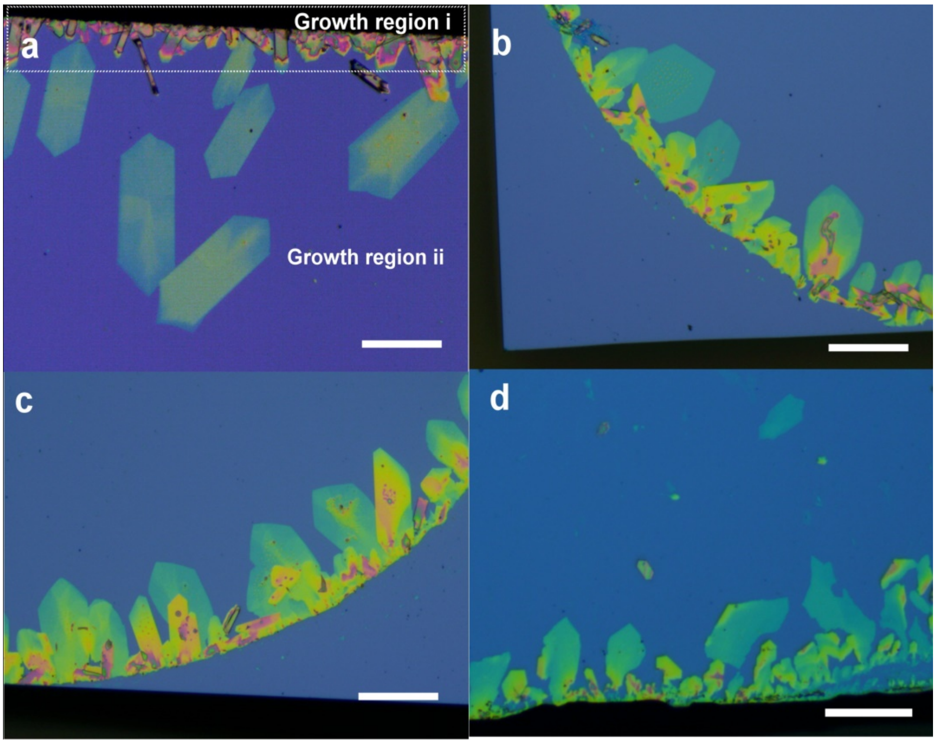

Supplementary Figure 18. The coffee ring effect in growth of p-MSB crystals. **a,** The two growth regions. Growth region i is located at the edge of the droplet or the substrate, where the growth takes place owing to the coffee ring effect. Growth region ii is located in the droplet, where the growth of 2D crystal takes place. **b-d,** The p-MSB crystals grown at the edge of the droplet or the substrate, owing to the coffee ring effect. Scale bars are 200 μm.

Supplementary Figure 19

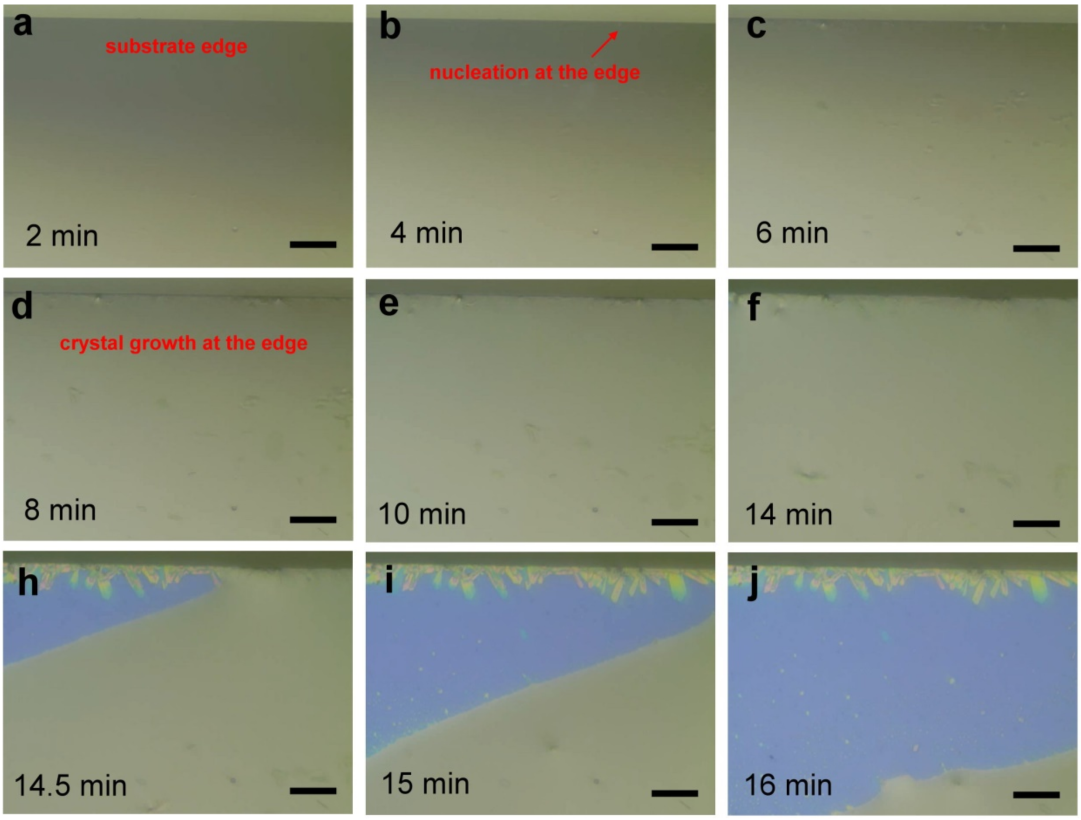

Supplementary Figure 19. In-situ observation of the crystal growth (toluene). **a-j,** The images were captured at 2 min, 4 min, 6 min, 8 min, 10 min, 14 min, 14.5 min, 15 min, 16 min, respectively, after the solution was dropped on SiO_2/Si. The growth takes place at the edge owing to the coffee ring effect. The scale bars are 200 μm.

Supplementary Figure 20

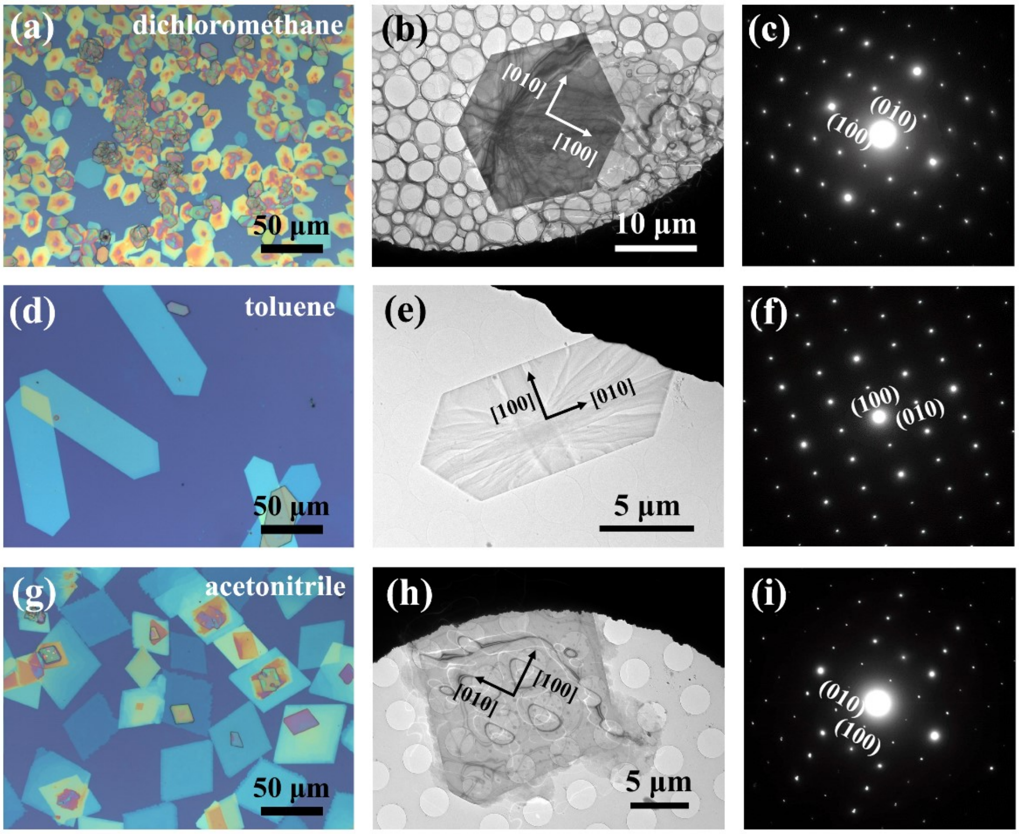

Supplementary Figure 20. 2D p-MSB crystals grown from different solvents. The optical and TEM images of 2D p-MSB crystals grown from different solvents of **a,b**, dichloromethane, **d,e,** toluene and **g,h,** acetonitrile at the fixed saturated concentration at room temperature. Crystals with different morphologies including hexagons, elongated hexagons and diamonds can be obtained by varying the solvent. **c,f,i,** The identical SAED patterns of different morphologies indicate that the as-grown crystals have the same crystalline structure.

Supplementary Figure 21

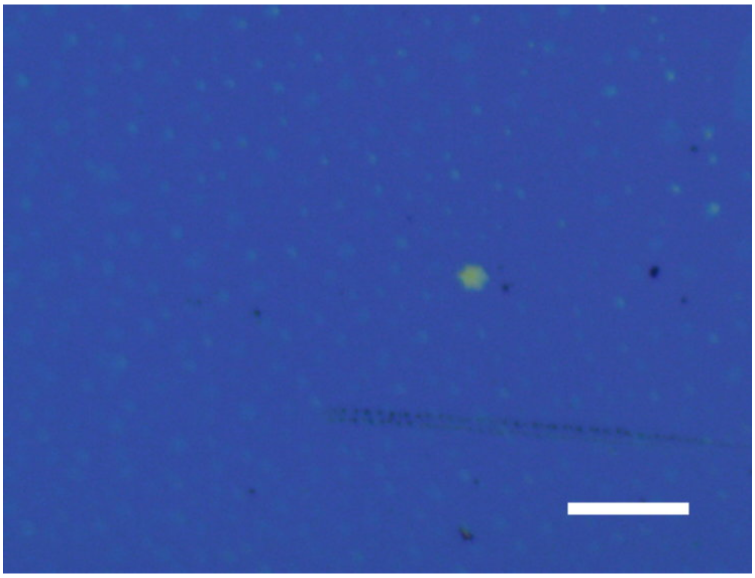

Supplementary Figure 21. Microscope image of the seed crystals. See details in Supplementary Note 6. The scale bars are 10 μm.

Supplementary Figure 22

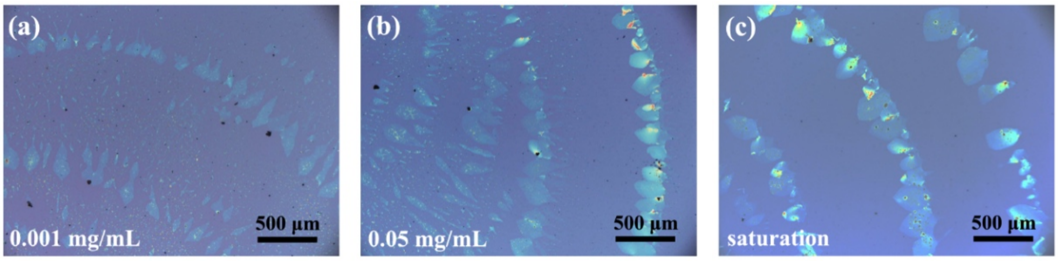

Supplementary Figure 22. The optical images of the coffee ring. The p-MSB samples are grown from toluene without seed crystals at room temperature and different solution concentrations of **a,** 0.001 mg mL^{-1}, **b,** 0.05 mg mL^{-1} and **c,** saturation, respectively. The concentric ring-like deposition was maintained as concentration gradually increased up to saturation.

Supplementary Figure 23

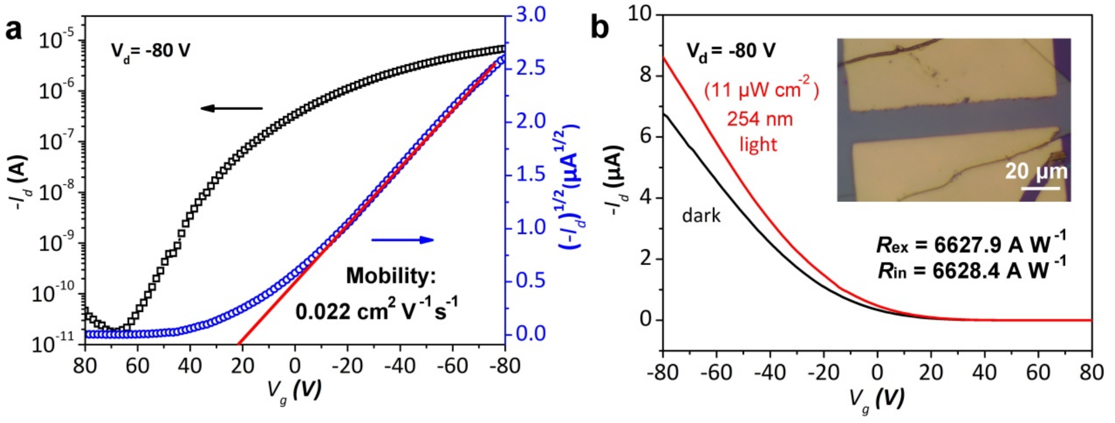

Supplementary Figure 23. A device based on a p-MSB single crystal (459 nm thick). The crystal is produced by physical vapor transport. **a,** The transfer curve of the device, from which the mobility is calculated to be 0.022 $cm^2\ V^{-1}\ s^{-1}$. **b,** The transfer curves in dark (black curve) and under 254 nm illumination (red curve, 11 $\mu W\ cm^{-2}$). R_{ex} and R_{in} are calculated to be 6627.9 A W^{-1} and 6628.4 A W^{-1} in the on-state of the device. The inset of **b** is the microscope image of the device.

Supplementary Figure 24

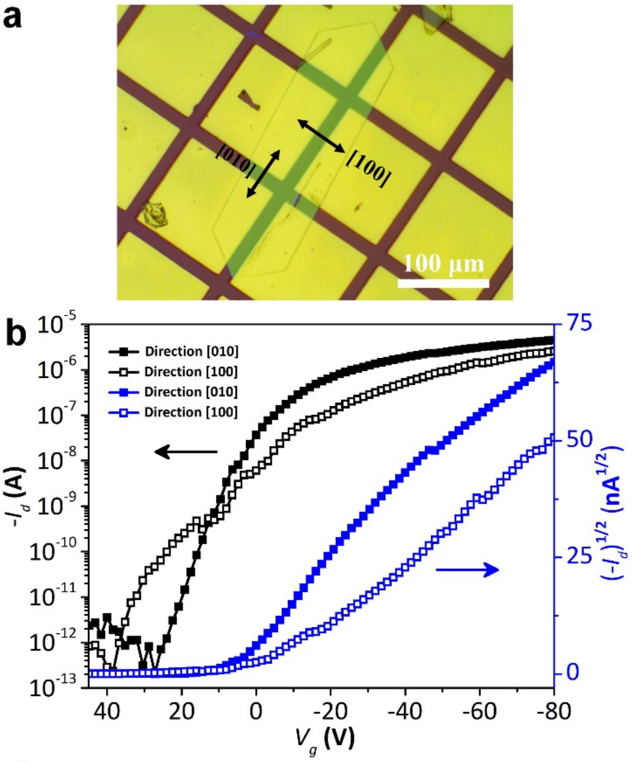

c

direction	μ (cm² V⁻¹ s⁻¹)	anisotropy
[010]	0.057	3.8
[100]	0.015	

Supplementary Figure 24. Anisotropic electrical property of a 2D p-MSB crystal. **a,** The optical image of the device. **b,** The transfer curves measured along different directions [100] and [010]. **c,** The hole mobility along [100] and [010]. The anisotropy is calculated to be 3.8.

Supplementary Figure 25

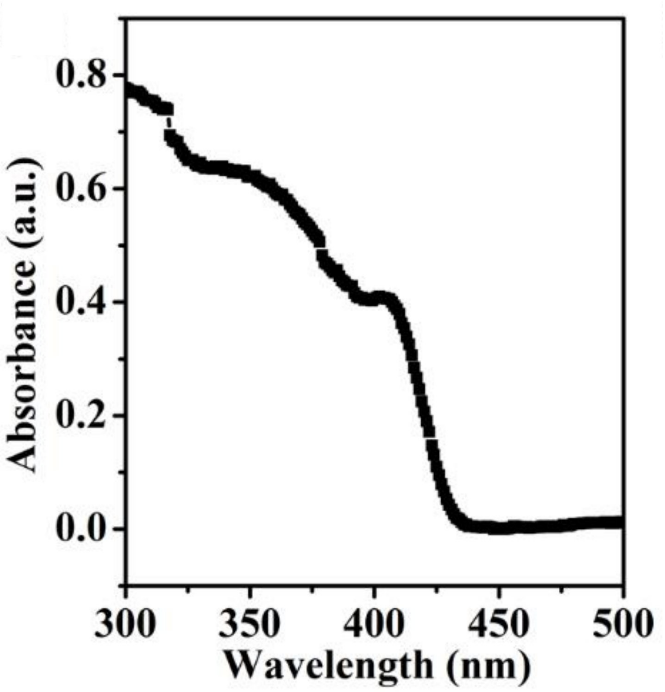

Supplementary Figure 25. UV-vis absorption spectrum of the 2D p-MSB crystal. The p-MSB crystal exhibits little light absorption beyond 420 nm.

Supplementary Figure 26

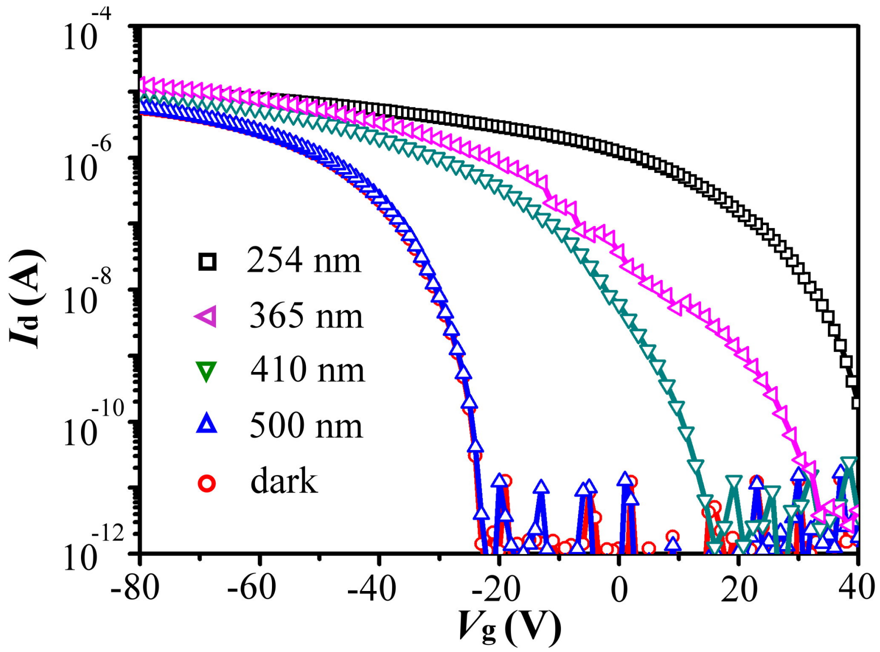

Supplementary Figure 26. The photoelectrical response of 2D p-MSB crystals. The device is measured under incident light with different wavelength. Transfer curves (V_d = −80 V) of a device based on a 2D p-MSB crystal in dark (red symbol) or under 43.1 μW cm^{-2} illumination at different wavelengths (244 nm, black symbol; 365 nm, purple symbol; 410 nm, green symbol; 500 nm, blue symbol).

Supplementary Figure 27

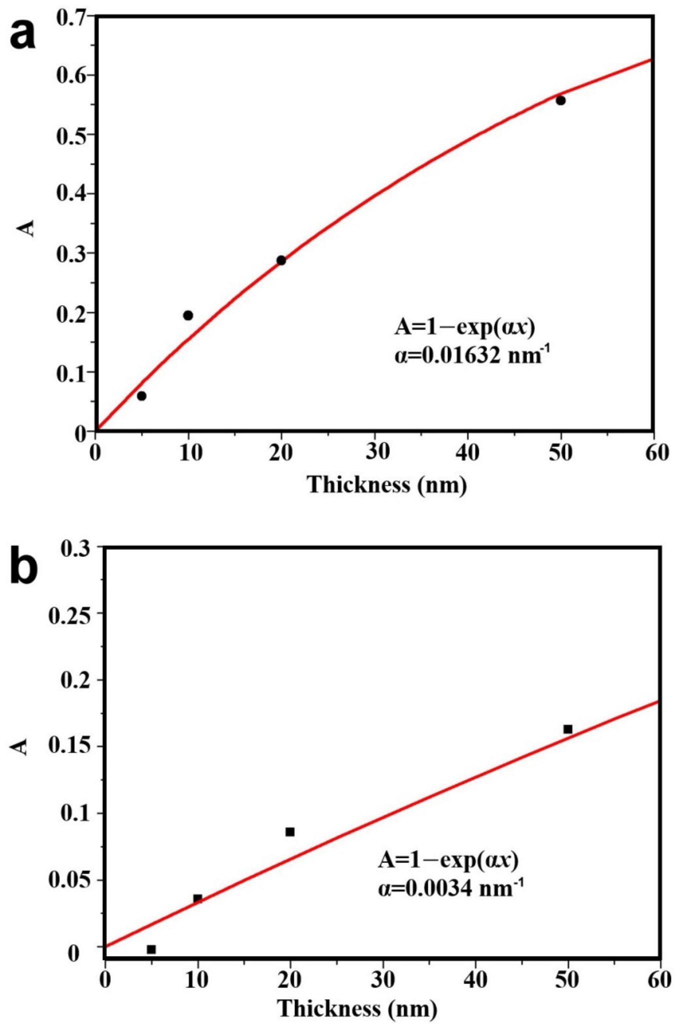

Supplementary Figure 27. The absorption vs. thickness. The absorption A of the p-MSB crystal on a quartz substrate with different thickness (solid squares) for 254 nm light (**a**) and 365 nm light (**b**). The red curves are the exponential fitting results by using the the Beer-Lambert Law: $A = 1 - \exp(\alpha x)$, from which the absorption parameters α are obtained to be 0.01632 nm^{-1} (254 nm) and 0.0034 nm^{-1} (365 nm).

Supplementary Figure 28

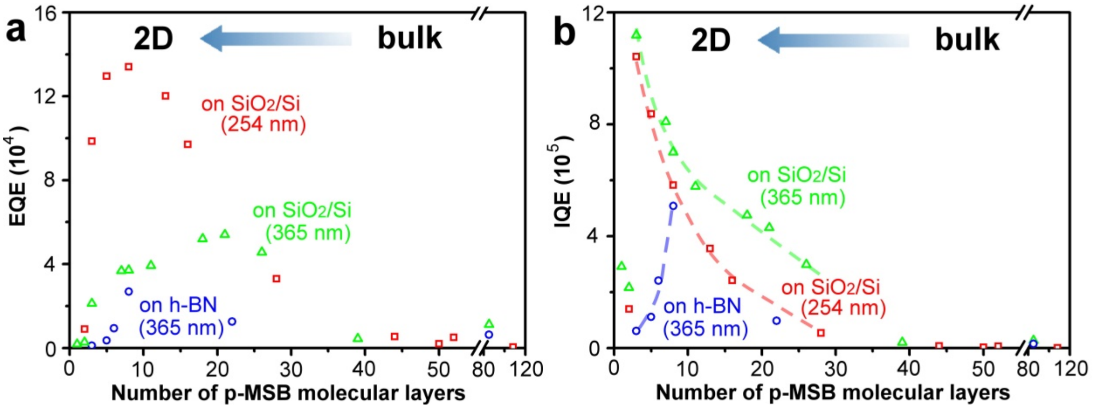

Supplementary Figure 28. Thickness dependence photoelectrical behavior. **a,** External quantum efficiency (EQE) and **b,** internal quantum efficiency (IQE) as a function of the number of p-MSB molecular layers under 14.1 μW cm^{-2} illumination at 365 nm or 12.1 μW cm^{-2} illumination at 254 nm.

Supplementary Figure 29

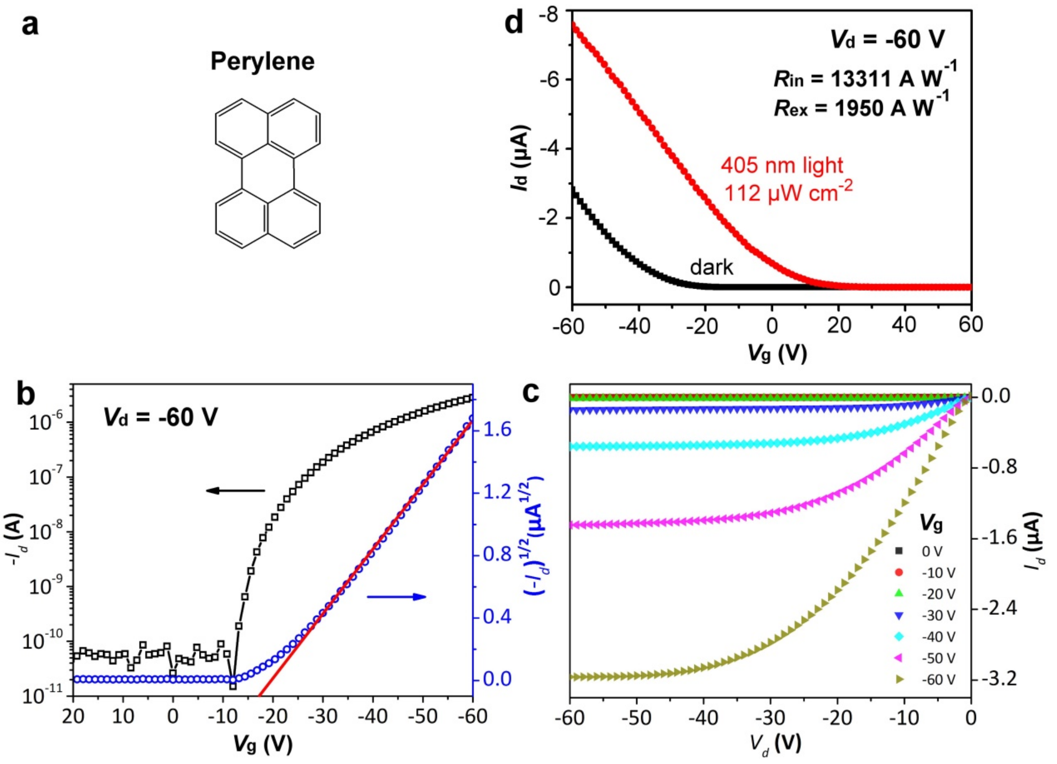

Supplementary Figure 29. Photoelectrical property of the perylene crystal. **a,** Molecular structure. **b,** Transfer (V_d = −60 V) and **c,** output curves of a Perylene (20 nm thick) field effect transistor. **d,** The transfer curves (V_d = −60 V) in dark or under 405 nm illumination (112 μW cm^{-2}). R_{in} and R_{ex} are calculated to be 13311 A W^{-1} and 1950 A W^{-1}, respectively.

Supplementary Figure 30

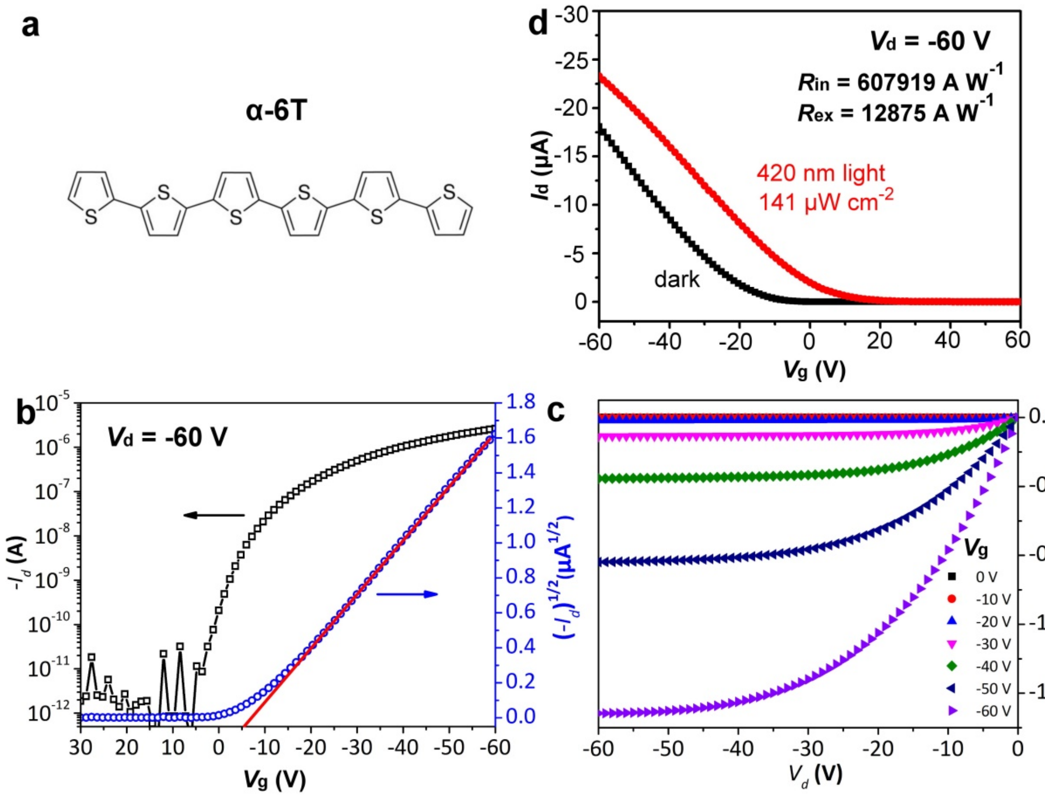

Supplementary Figure 30. Photoelectrical property of the α-sexithiophene crystal. **a,** Molecular structure. **b,** Transfer (V_d = −60 V) and **c,** output curves of a α-sexithiophene (α-6T, 10 nm thick) field effect transistor. **d,** The transfer curves (V_d = −60 V) in dark or under 420 nm illumination (141 μW cm^{-2}). R_{in} and R_{ex} are calculated to be 607919 A W^{-1} and 12875 A W^{-1}, respectively.

Supplementary Figure 31

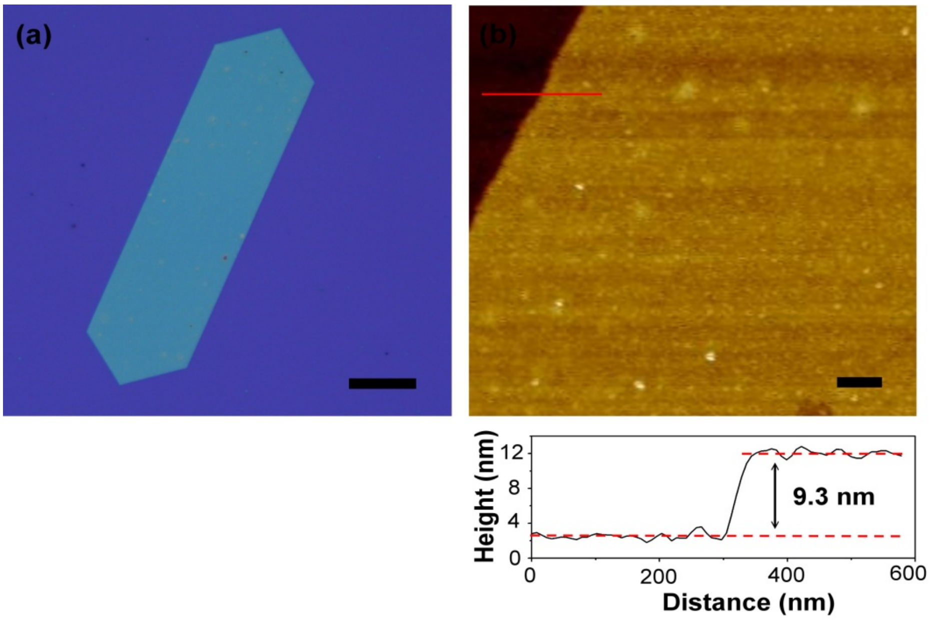

Supplementary Figure 31. The 2D p-MSB crystal grown on *h*-BN. **a,** Optical microscope image and **b,** AFM image of a 2D p-MSB crystal on *h*-BN. The *h*-BN film is grown on the SiO_2/Si by plasma enhanced chemical vapor deposition. The scale bars are 50 μm in **a** and 200 nm in **b**. The inset is the height profile along the red line in **b**.

Supplementary Figure 32

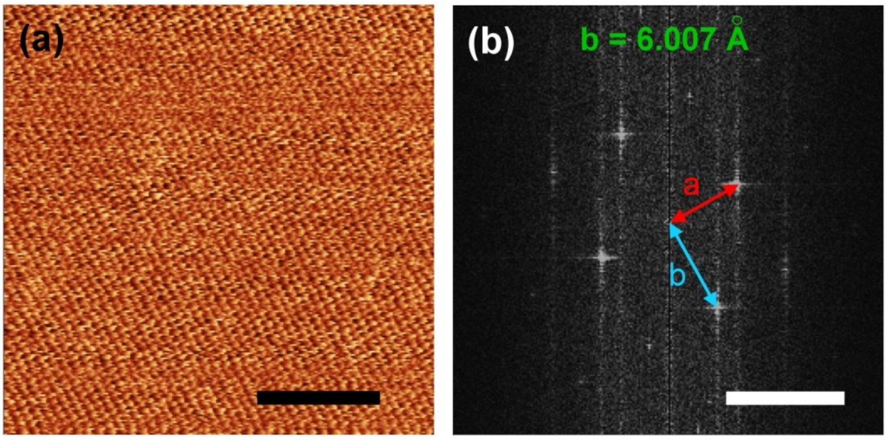

Thickness: bulk (110 nm)

a/nm^{-1}	1.329 ± 0.003	a/Å	7.52 ± 0.02
b/nm^{-1}	1.665 ± 0.004	b/Å	6.01 ± 0.01

Supplementary Figure 32. AFM characterization of a bulk p-MSB crystal. The sample is (thickness: 110 nm) grown on h-BN. **a,** High-resolution AFM image and **b,** FFT pattern of a p-MSB crystal on h-BN. Lattice parameters is calculated from the FFT pattern. The h-BN film is grown on the SiO_2/Si by plasma enhanced chemical vapor deposition. The scale bars are 10 nm in **a** and 2 nm^{-1} in **b**.

Supplementary Figure 33

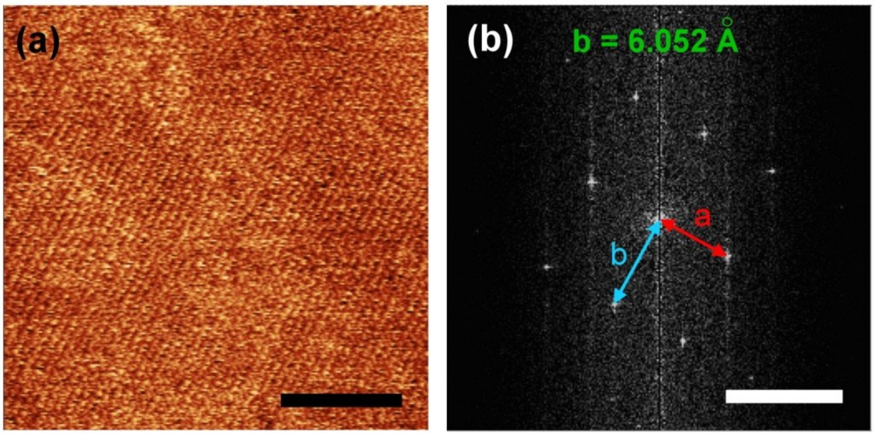

Thickness: few-layer (5.7 nm)

a/nm^{-1}	1.328 ± 0.006	a/Å	7.53 ± 0.04
b/nm^{-1}	1.652 ± 0.005	b/Å	6.05 ± 0.02

Supplementary Figure 33. AFM characterization of a few-layer p-MSB crystal. The crystal is (thickness: 5.7 nm) grown on h-BN. **a,** High-resolution AFM image and **b,** FFT pattern of a p-MSB crystal on h-BN. Lattice parameters is calculated from the FFT pattern. The h-BN film is grown on the SiO_2/Si by plasma enhanced chemical vapor deposition. The scale bars are 10 nm in **a** and 2 nm^{-1} in **b**.

Supplementary Figure 34

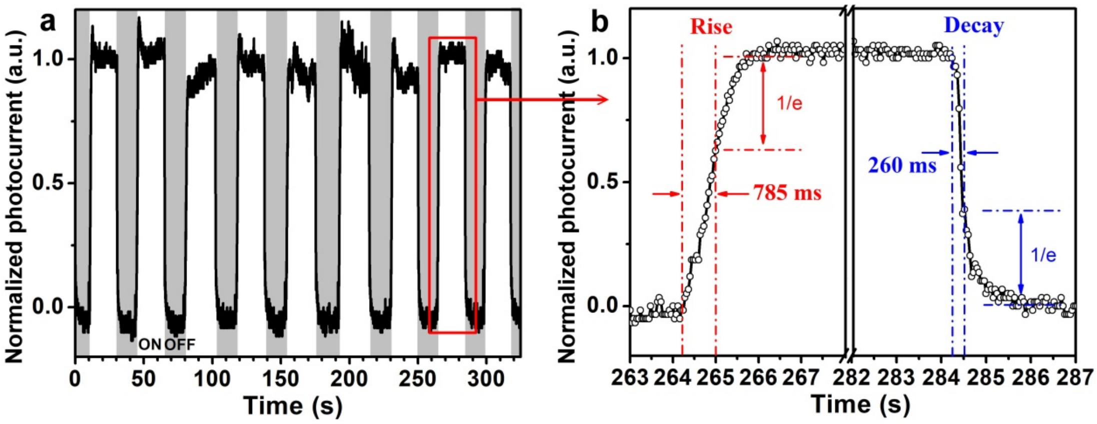

Supplementary Figure 34. The dynamic photocurrent response. **a,** The normalized photocurrent of the 2D p-MSB device when the light was turned on and off ($V_d = -80$ V, $V_g = 0$ V). **b.** A circle of normalized photocurrent response.

Supplementary Figure 35

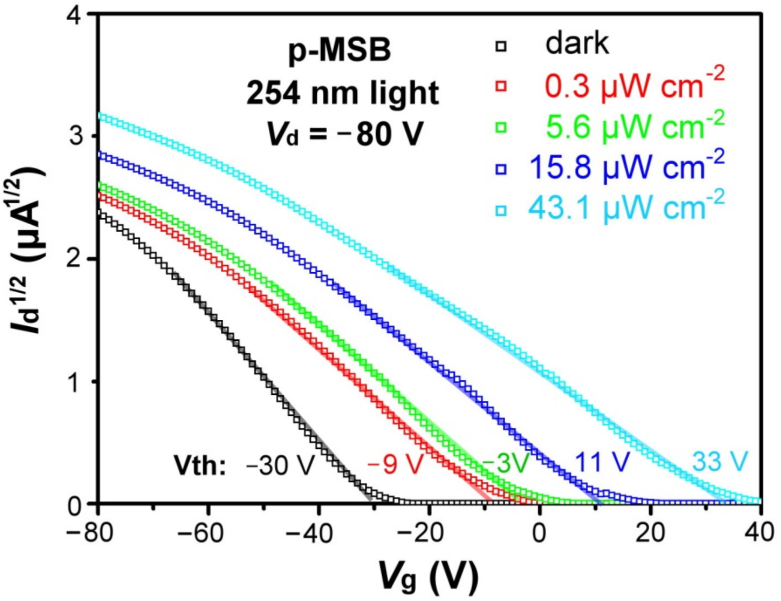

Supplementary Figure 35. Transfer curves of a p-MSB device (V_d = −80 V). The device is in the dark or under 254 nm illumination with different power intensities. The threshold voltage shifts from ~ −30 V to ~33 V when the illumination power density increases from dark to 43.1 μW cm^{-2}.

Supplementary Tables

Supplementary Table 1

Layer	Total thickness(nm)	Layer thickness (nm)
1	2.2 ± 0.2	2.2
2	4.3 ± 0.3	2.1
3	6.4 ± 0.2	2.1
4	8.4 ± 0.2	2.0
5	10.4 ± 0.6	2.0
6	12.3 ± 0.3	1.9
…	…	…
85	162.3 ± 0.6	1.9

Supplementary Table 1. Total thickness of different p-MSB single crystals on SiO_2/Si, and the thickness of each layer.

Supplementary Table 2

Layer	Total thickness(nm)	Layer thickness (nm)
3	3.8 ± 0.3	
4	5.6 ± 0.3	1.8
5	7.4 ± 0.2	1.8
6	9.3 ± 0.4	1.9
5	11.2 ± 0.2	1.9

Supplementary Table 2. Total thickness of different p-MSB single crystals on h-BN, and the thickness of each layer.

Supplementary Notes

Supplementary Note 1. In-situ observation of the 2D crystal growth.

To clarify the growth mechanism, we observed the growth of 2D p-MSB crystals by using optical microscope. After heating the oversaturated toluene solution to 70 °C, a drop of solution (5 μL) containing seed crystals was poured over a cleaned SiO_2/Si substrate placed inside an uncovered Petri dish under ambient atmosphere. At the same time, the growth process was captured by an optical microscope (Olympus, 5× object lens). The video was captured under microscope illumination and the crystal growth took place in an uncovered Petri dish, thus the solvent was evaporated within several minutes, faster than the actual growth process.

Although this process was faster and the contrast of the crystal in the solution was lower than that on bare SiO_2/Si, we still in-situ observed the growth process of the 2D p-MSB crystal (Supplementary Movie 1, Supplementary Fig. 10). In the actual growth process with slower solvent evaporation rate and higher temperature environment, 2D p-MSB crystals with smaller thickness and larger size were expected. The growth of 2D crystal mainly took place on the SiO_2/Si surface within the toluene solution. The crystal size gradually increased along with the solvent evaporation without obvious contrast change, indicating a 2D growth mode. In the video, we observed some thick p-MSB crystals grown on the solution surface, which should be attributed to the surface nucleation due to the fast solvent evaporation. With slower evaporation in a covered Petri dish, these thick crystals grown on the solution surface will disappear.

Supplementary Note 2. The external condition for 2D p-MSB growth.

Although the actual solution-based growth mechanism is complicated, controlled

experiments show that a steady thermodynamically controlled growth process is required for the 2D p-MSB crystal growth inside the solution. Such a thermodynamically controlled growth requires relatively high growth temperature, oversaturated solution with high boiling solvent and seed crystals. As shown in Fig. 3i, the growth of 2D p-MSB crystal inside the solution takes place at 373 K by using an oversaturated toluene solution with seed crystals (p-MSB concentration: 0.2 mg mL^{-1}). To achieve a slow and steady evaporation of the solvent, the substrate is placed inside a covered Petri dish under ambient atmosphere. To maintain the temperature, the Petri dish is on a hot plate.

The 2D crystal growth is sensitive to external conditions, including the growth temperature, evaporation rate, seed crystals, the solvent boiling point, viscosity and surface tension of the solvent, etc. Without a thermodynamically controlled growth condition, disordered 3D crystals or amorphous samples are produced.

Different growth temperature: Supplementary Note 4 and Fig. 3d show that the crystal size decreases and the thickness increases with decreasing the growth temperature. If we cooled the solution by ice bath in the growth, only small thick p-MSB crystals were obtained (Supplementary Fig. 14).

Different amount of seed crystals: Without the seed crystals, we cannot observe the 2D crystal growth inside the solution, while some crystals grow at the edge of solution owing to the coffee ring effect (Supplementary Fig. 18, Supplementary Movie 2). If we use an oversaturated toluene solution with a large amount of seed crystals, we can only prepare small p-MSB crystals (Supplementary Fig. 11d).

Different solvent boiling point: If we use the solvent with low boiling point such as dichloromethane and acetonitrile, the solvent evaporates rapidly, resulting in large amounts of small crystals or nuclei (Supplementary Fig. 20).

Different solvent viscosity and surface tension: The growth of the p-MSB inside

the solution requires a solvent with small viscosity and surface tension[1]. Otherwise, the growth takes place at the solution/air interface or droplet edge. See details in Supplementary Note 3.

Different evaporation rate: A slow and steady evaporation rate is required for the growth of 2D p-MSB crystals inside the solution. If we quickly dried the toluene solvent by heating in an uncovered Petri dish (Supplementary Fig. 11a, b) or using nitrogen gas (Supplementary Fig. 11c), only some amorphous or non-uniform structures were obtained.

Supplementary Note 3. Effect of solvent viscosity and surface tension.

The solvent viscosity and surface tension can influence the solution-based crystal growth process. According to recent literature[1], 2D crystals tend to grow at the liquid-air interface in the case of a solvent with high viscosity and surface tension, while 3D crystals are obtained if the growth takes place inside the solution in the case of a solvent with low viscosity and surface tension.

In the control experiments, we used solvents with different viscosity and surface tension, including dimethyl phthalate (DMP, viscosity: 14.4 mPa*s, surface tension: 41.9 mN/m), dimethyl sulfoxide (DMSO, 4 mPa*s, surface tension: 43.5 mN/m), toluene (0.6 mPa*s, surface tension: 28.6 mN/m). Similar with the results in the literature, the p-MSB crystal or film grows mainly at the liquid-air interface or the droplet edge (Supplementary Fig. 12, 13) in the case of the solvent with high viscosity and surface tension (DMP, DMSO), while the growth takes place inside the solution (Supplementary Fig. 10, Supplementary Video 1) in the case of the solvent with low viscosity and surface tension (toluene). However, the growth of 2D crystal is not only determined by the location where the growth takes place. For instance, the crystals grown inside the toluene solution have smaller thickness and better uniformity, compared with that grown at the liquid-air interface of toluene (Supplementary Fig.

10), at the liquid-air interface of DMP (Supplementary Fig. 12f) or at the droplet edge of toluene (Supplementary Fig. 18). Thus, other factors also influence the growth of 2D crystals. In fact, previous literatures[1-4] have developed technologies and investigated the factors to produce 2D organic crystals at the liquid-air interface. This work aims to realize 2D crystal growth inside the solution, which usually obtains 3D crystals as demonstrated in previous literature[1], and finds that the growth of 2D crystals inside the solution requires a thermodynamically controlled condition.

Supplementary Note 4. The p-MSB crystal growth at different temperature

In the experiments, we kept the covered Petri dish with the growth substrate on a hot plate to maintain the growth temperature, after dropping the hot oversaturated solution onto the growth substrate. Supplementary Fig. 14a shows a substrate after crystal growth at 353 K. We can observe large area 2D crystals with size up to 2.5 mm on the substrate. We also tried to produce p-MSB crystals without extra heat source. In ambient condition, the hot solution was gradually cooled to room temperature. After growth, the crystals were normally smaller than the samples grown on the hot plate (Supplementary Fig. 14b). If we dropped room temperature oversaturated solution onto the growth substrate, some thick crystals with small size (100~200 μm, Supplementary Fig. 14c) were obtained after growth. If we cooled down the solution by using ice bath in the growth, only small 3D crystals (< 30 μm) were obtained (Supplementary Fig. 14d). Therefore, higher solution temperature is beneficial to the growth of large sized 2D crystals.

Supplementary Note 5. Coffee ring effect in the crystal growth.

Coffee ring phenomenon is commonly observed during evaporating droplet

containing nonvolatile solutes resulted from a combination of capillary flow and pinning of the contact line[5]. When the contact line of a drying droplet is pinned, the liquid evaporating from the edge is replenished by the liquid from the interior, so the outward flow carries entrained solutes to the drop periphery. As a result, higher concentration is expected, leading to deposition at the three-phase contact interface near the edge of the solution[5-7]. A repeated pinning-depinning process usually leads to successive coffee-ring-like structures. The coffee ring effect normally exists in the solution-based process of organic materials, leading to the nucleation and growth of organic crystals at the edge of the droplet[5,6].

Although the 2D p-MSB crystals were mainly obtained inside the solution, we still observed some p-MSB crystals grown at the edge of substrate or droplet (Supplementary Fig. 18) owing to the coffee ring effect. As a result of the capillary flow during the solvent evaporation, the nucleation and growth of p-MSB crystals not only take place on the seed crystals but also at the edge of the substrate or the droplet. Supplementary Fig. 19 and Supplementary Movie 2 show the growth process of p-MSB crystals attributed to the coffee ring effect.

If we used unsaturated or saturated toluene solution without seed crystals, the nucleation and growth only took place at the edge of the substrate or the droplet. As a result, no 2D p-MSB crystals with elongated hexagonal shapes were obtained. The products were concentric rings of flakes (Supplementary Fig. 22) which were attributed to the coffee ring effect as well as the repeated stick-slip motion of contact line (Supplementary Movie 3). Although the seed crystals are hard to be in-situ characterized in the solution by optical microscope, this result reveals the significant role of the seed crystals in the growth of 2D p-MSB crystals within solution.

Supplementary Note 6. Experimental details of the preparation of the seed crystals

To prepare oversaturated solution with seed crystals, 2.0 mg p-MSB (98%, TCI Chemicals) was added in 10 mL toluene, and was subjected in an ultrasound bath at 60 °C for 30 minutes to achieve the complete dissolution. After that, the oversaturated solution was placed in a sealed container, and was then slowly cooled down to room temperature without agitation for about 30 minutes. Some seed crystals formed in the solution. The solution with seed crystals was heated to 60 °C for 5 minutes and then was slowly cooled down to room temperature without agitation for about 30 minutes. The heating and cooling process was repeated for 2~3 time. Finally, the oversaturated solution with seed crystals was obtained for the growth of 2D crystals.

It was hard to in-situ and real-time characterize the seed crystals as a result of the small size, weak contrast in the solution and fast transformation with the surrounding. To observe the seed crystals, a clean SiO_2/Si substrate was dipped into the hot oversaturated solution with seed crystals, and then was blown dry using a N_2 gun to remove the solution. Some crystals were adsorbed on the SiO_2/Si, which were characterized by using optical microscope (Olympus).

Supplementary Note 7. Detail discussion of the photo-gating effect

Photo-gating effect is attributed to the prolonged excess carrier lifetime induced by material surface, defects, impurities, artificial designed interfaces or hybrid structures. If one type of the photo-generated carriers is trapped, they can serve as an additional electric field like gate voltage to shift the threshold voltage, thus modulating the channel conductance and introducing extra increase of the photoresponsivity[8]. Owing to the large surface-to-volume ratio and reduced screening, large photo-gating effect normally exists in low dimensional materials[8]. The lifetime of the trapped charge is equal to the time needed for photocurrent to drop to its 1/e in the recombination process[9]. According to normalized photocurrent vs. response time curve (Supplementary Fig. 34), the lifetime of the trapped electrons in the 2D p-MSB

crystal is 260 ms. The long lifetime, which might be related to the weak binding between electron-hole pairs, indicates an efficient electron trapping capability of the 2D p-MSB crystal[10]. Owing to the back-gate electrical field, the confinement of holes in a narrow region leads to a spatial separation between the transporting holes and the trapped electrons, thus reducing the electron-hole recombination and increasing the density of trapped electrons[10]. The density of the trapped electrons (Δn) can be calculated by[11]

$$\Delta n = C_{ox}\,\Delta V_{th} / q \qquad (1)$$

Where the $C_{ox} = 1.2\times10^{-8}$ F cm^{-2} is the gate capacitance of the ~300 nm thick SiO_2, ΔV_{th} is the shift of the threshold voltage, q is the electron charge. Thus, according to the transfer curves under 254 nm illustration (Supplementary Fig. 35), the density of the trapped electrons is about 1.57×10^{12} cm^{-2}, 2.03×10^{12} cm^{-2}, 3.08×10^{12} cm^{-2}, 4.73×10^{12} cm^{-2}, when the power density of the 254 light is 0.3 μW cm^{-2}, 5.6 μW cm^{-2}, 15.8 μW cm^{-2}, 43.1 μW cm^{-2}, respectively.

Supplementary Note 8. Calculation of the light absorbance

The absorbance was calculated by the Beer-Lambert Law. The Beer-Lambert law is the linear relationship between the absorbance and the concentration as well as the thickness of an absorbing species. However, considering that the incident light gradually decreases along the path length, the absorbance is non-linear with the thickness of the absorbing species. The relationship of the absorbance and the thickness should be[9]:

$$A = 1 - \exp(-\alpha x) \qquad (2)$$

where A is the absorbance, α is the absorption parameter of the absorbing species, x is the thickness. According to this equation (Supplementary Equation 2), A is about 1 for bulk samples with extra-large thickness. In the experiments, we measured the

absorbance of 2D p-MSB crystals with different thicknesses (Supplementary Fig. 27). The red lines are the fitting curves by using this equation (Supplementary Equation 2), from which the absorption parameter α of p-MSB crystal is obtained to be 0.01632 nm^{-1} (254 nm) and 0.0034 nm^{-1} (365 nm). And then, we can calculate the absorbance of the p-MSB crystals with different thickness by using this equation (Supplementary Equation 2) and the α values. IQE could be calculated as IQE = EQE / A, while the R_{in} can be calculated by $R_{in} = R_{ex} / A$.

Supplementary References

1. Vladmirov, I. et al. High-mobility, ultrathin organic semiconducting films realized by surface-mediated crystallization. *Nano Lett.* **18,** 9−14 (2018).

2. Wang, Q. et al. 2D single-crystalline molecular semiconductors with precise layer definition achieved by floating-coffee-ring-driven assembly. *Adv. Funct. Mater*. **26,** 3181−3181 (2016).

3. Xu, C. et al. A general method for growing two-dimensional crystals of organic semiconductors by "solution epitaxy". *Angew. Chem. Int. Ed*. **55,** 9519−9523 (2016).

4. Diao, Y. et al. Solution coating of large-area organic semiconductor thin films with aligned single-crystalline domains. *Nat. Mater.* **12,** 665−671 (2013).

5. Han, W. & Lin, Z. Learning from "coffee rings": ordered structures enabled by controlled evaporative self-assembly. *Agnew. Chem. Int. Ed.* **51,** 1534−1546 (2015).

6. Bi, S., He, Z., Chen, J. & Li, D. Solution-grown small-molecule organic semiconductor with enhanced crystal alignment and areal coverage for organic thin film transistors. *AIP Adv.* **5,** 077170 (2015).

7. Zhang, L et al. Inkjet printing high-resolution, large-area graphene patterns by coffee-ring lithography. *Adv. Mater.* **24,** 436–440 (2012).

8. Fang, H. & Hu, W. Photogating in low dimensional photodetectors. *Adv. Sci.* **4,** 1700323 (2017).

9. Liu, X. et al.. Epitaxial ultrathin organic crystals on graphene for high-efficiency phototransistors. *Adv. Mater*. **28,** 5200−5205 (2016).

10. He, X. et al. Photogenerated intrinsic free carriers in small-molecule organic semiconductors visualized by ultrafast spectroscopy. *Sci. Rep.* **5,** 17076 (2015).

11. Yamamoto, M., Ueno, Keiji & Tsukagoshi, K. Pronounced photogating effect in atomically thin WSe_2 with a self-limiting surface oxide layer. *Appl. Phys. Lett.* **112,** 181902 (2018).
