# Supplementary material for: Enhanced photoelectrical response of thermodynamically epitaxial organic crystals at the two-dimensional limit
Source: Nat Commun. 2019 Feb 14;10:756. doi: 10.1038/s41467-019-08573-8 (PMC6375977; doi:10.1038/s41467-019-08573-8)
Supplement: Supplementary file 2 — Description of Additional Supplementary Files [file 41467_2019_8573_MOESM2_ESM.pdf]

Description of Additional Supplementary Files

File Name: Supplementary Movie 1
Description: The growth of 2D p-MSB crystal.

File Name: Supplementary Movie 2
Description: The formation of coffee ring.

File Name: Supplementary Movie 3
Description: The formation of coffee ring.
